# Multivariate Metal–Organic Framework/Single-Walled Carbon Nanotube Buckypaper for Selective Lead Decontamination

**DOI:** 10.1021/acsanm.2c00280

**Published:** 2022-04-01

**Authors:** Mariafrancesca Baratta, Teresa Fina Mastropietro, Rosaria Bruno, Antonio Tursi, Cristina Negro, Jesús Ferrando-Soria, Alexander I. Mashin, Aleksey Nezhdanov, Fiore P. Nicoletta, Giovanni De Filpo, Emilio Pardo, Donatella Armentano

**Affiliations:** † Dipartimento di Chimica e Tecnologie Chimiche (CTC), Università della Calabria, Rende 87036, Cosenza, Italy; ‡ Instituto de Ciencia Molecular (ICMol), Universidad de Valencia, 46980 Paterna, Valencia, Spain; ◊ Applied Physics & Microelectronics, Lobachevsky State University of Nizhni Novgorod, 603022 Nizhni Novgorod, Russian Federation; # Dipartimento di Farmacia e Scienze della Salute e della Nutrizione, Università della Calabria, 87036 Rende, Italy

**Keywords:** single-walled carbon nanotube membranes, multivariate metal−organic frameworks, lead decontamination, MOF-based composites, water remediation

## Abstract

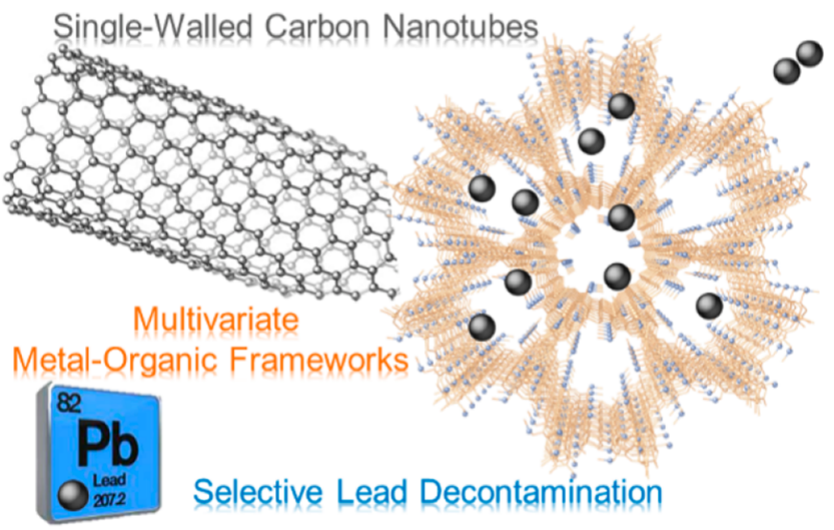

The search for efficient technologies empowering the selective capture of environmentally harmful heavy metals from wastewater treatment plants, at affordable prices, attracts wide interest but constitutes an important technological challenge. We report here an eco-friendly single-walled carbon nanotube buckypaper (SWCNT-BP) enriched with a multivariate amino acid-based metal–organic framework (MTV-MOF) for the efficient and selective removal of Pb^2+^ in multicomponent water systems. Pristine MTV-MOF was easily immobilized within the porous network of entangled SWCNTs, thus obtaining a stable self-standing adsorbing membrane filter (**MTV-MOF/SWCNT-BP**). SWCNT-BP alone shows a moderately good removal performance with a maximum adsorption capacity of 180 mg·g^–1^ and a considerable selectivity for Pb(II) ions in highly concentrated multi-ion solutions over a wide range of lead concentration (from 200 to 10000 ppb). Remarkably, these features were outperformed with the hybrid membrane filter **MTV-MOF/SWCNT-BP**, exhibiting enhanced selectivity and adsorption capacity (310 mg·g^–1^, which is up to 42% higher than that of the neat SWCNT-BP) and consequently enabling a more efficient and selective removal of Pb^2+^ from aqueous media. **MTV-MOF/SWCNT-BP** was able to reduce [Pb^2+^] from the dangerous 1000 ppb level to acceptable limits for drinking water, below 10 ppb, as established by the current EPA and WHO limits. Thus, the eco-friendly composite **MTV-MOF/SWCNT-BP** shows the potential to be effectively used several times as a reliable adsorbent for Pb^2+^ removal for household drinking water or in industrial treatment plants for water and wastewater lead decontamination.

## Introduction

Access to safe drinking water is vital for both human life and general public health, as contamination of aquatic environments increases the transmission of life-threatening diseases.^1^ Among the wide plethora of organic and inorganic pollutants aquatic environments may contain, which are currently regulated by national and international agencies worldwide,^2−4^ water contamination from lead has received particular attention.^2−4^ Lead contamination of water sources arises from diverse human activities. For example, some manufacturing and industrial facilities still employ lead in many processes, releasing this toxic metal ion into wastewater at high concentrations.^5,6^ However, the main sources of domestic water lead-contamination are the corrosion of household plumbing systems or the erosion of natural deposits.

Exposure to lead has detrimental effects on aquatic ecosystems and human health, as a consequence of its bioaccumulation and the severe diseases it can cause even at minor concentration (>0.4 mg L^–1^).^7,8^ For instance, the maximum contaminant level goal (MCLG), established by the United States Environmental Protection Agency (EPA) through the Safe Drinking Water Act (SDWA),^9^ is 0 μg/L. However, it is extremely difficult to reach such ideal concentration by phosphate dosing, which is the most common technique used to prevent water contamination from lead. In this sense, the maximum contaminant level (MCL) that can be achieved using the best affordable available treatment technologies is fixed to 15 μg/L (15 ppb), whereas the maximum allowed levels for lead, established by the EPA^10^ and the U.S. Food and Drug Administration (FDA),^11^ are 10 μg/L and 5 μg/L (for bottled water), respectively.

A recent tragic example of lead contamination of aquatic environments can be found in Flint (Michigan, United States). The Flint water crisis (2014–2019) was a public health crisis which originated when the Flint River was contaminated with high levels of lead because of excessive pipe corrosion. Pb^2+^ concentration greatly exceeded the established limit and reached very hazardous levels (>5000 μg/L) in several water samples. The highest observed concentration was of 13200 μg/L at the so-called Resident Zero’s home.^12^ Because this contamination problem would likely have gone undetected indefinitely, without the investigation of Resident Zero, it has renewed the urgency for optimized corrosion controls, as well as for adopting advanced purification systems to prevent similar catastrophic situations in the future, especially in low-incoming countries with old and poorly maintained infrastructures.^13^

Several different techniques have been proposed for Pb(II) removal from aqueous systems, such as chemical precipitation, electrochemical procedures, solvent extraction, ion exchange, flocculation, nanofiltration, and solid-phase adsorption on a wide variety of inorganic and organic materials.^14−16^ However, a definitive technique enabling the reduction of Pb^2+^ to safe acceptable limits in contaminated water has not been fully implemented yet.

Metal–organic frameworks (MOFs)^17^ are hybrid porous materials that have already shown great efficiency in the removal of both organic^18−21^ and inorganic^22−24^ contaminants. They combine water stability and highly and well-defined functional^25,26^ porous structures with large internal surface areas, which allow for tailorable host–guest interactions and consequently enhanced affinity for target contaminants.^27^ In particular, a few water-stable MOFs and MOF-based nanocomposites have been investigated as potential Pb^2+^ adsorbents.^28−40^ Some of them show high adsorption capacities^40^ and selectivities,^39^ even in multicomponent metal ion water systems and/or in the presence of foulants. However, despite these remarkable advances, to the best of our knowledge there is no reported MOF able to clean up contaminated waters within limits accepted for drinking water (10 ppb, 5 ppb for bottled water). Another relevant aspect to be considered is the structuration/process of MOFs powders into more manageable materials.^41^ The mass production and large-scale application of MOF adsorbents and nanoadsorbent materials as fine powders are often problematic from a technical point-of-view and very expensive. Also, they present hazard concerns regarding their not-friendly manipulation and likely accidental release into the environment. Thus, the development of structured MOF materials in the form of scalable, and if possible cheap, beads, granules, and membranes is a required step toward real-world applications.^42−44^

Recently, the fabrication of multivariate MOFs^45−49^ (MTV-MOFs), possessing different and controlled functional groups within their channels, have emerged as suitable materials aiming at fabricating modular materials containing cooperative functionalities that can act synergistically to capture contaminants of very different natures. As a proof of concept, some of us recently reported outstanding examples of MTV-MOFs^50,51^ with environmental application in water remediation. In particular, one of them was reported to simultaneously and efficiently remove both inorganic heavy metals and organic dyes.^51^ Although it is quite well-established that the presence of functional groups (amino, thiol, and oxygen-containing groups) decorating MOFs channels is valuable for effective Pb^2+^ removal, much work remains to be done in order to explore, in detail, the interaction mechanisms involved in the capture process. To this end, single-crystal X-ray crystallography (SCXRD) is an extraordinary tool, but it is not frequently used in the elucidation of host–guest interactions for water remediation applications as would be desirable.

Single-walled carbon nanotube buckypapers (SWCNT-BPs) have emerged as very versatile materials in a wide range of applications. For example, they have been revealed as interesting materials for fire protection, heat dispersion systems in microelectronics, TV screens, electromagnetic interferences shielding systems, electrical-conductive tissues, photocatalytic substrates, electrodes for batteries, and supercapacitors.^52^ More recently, SWCNT-BPs have been proposed as innovative high-temperature-resistant and lightweight filtration systems.^53,54^

**Scheme 1:**
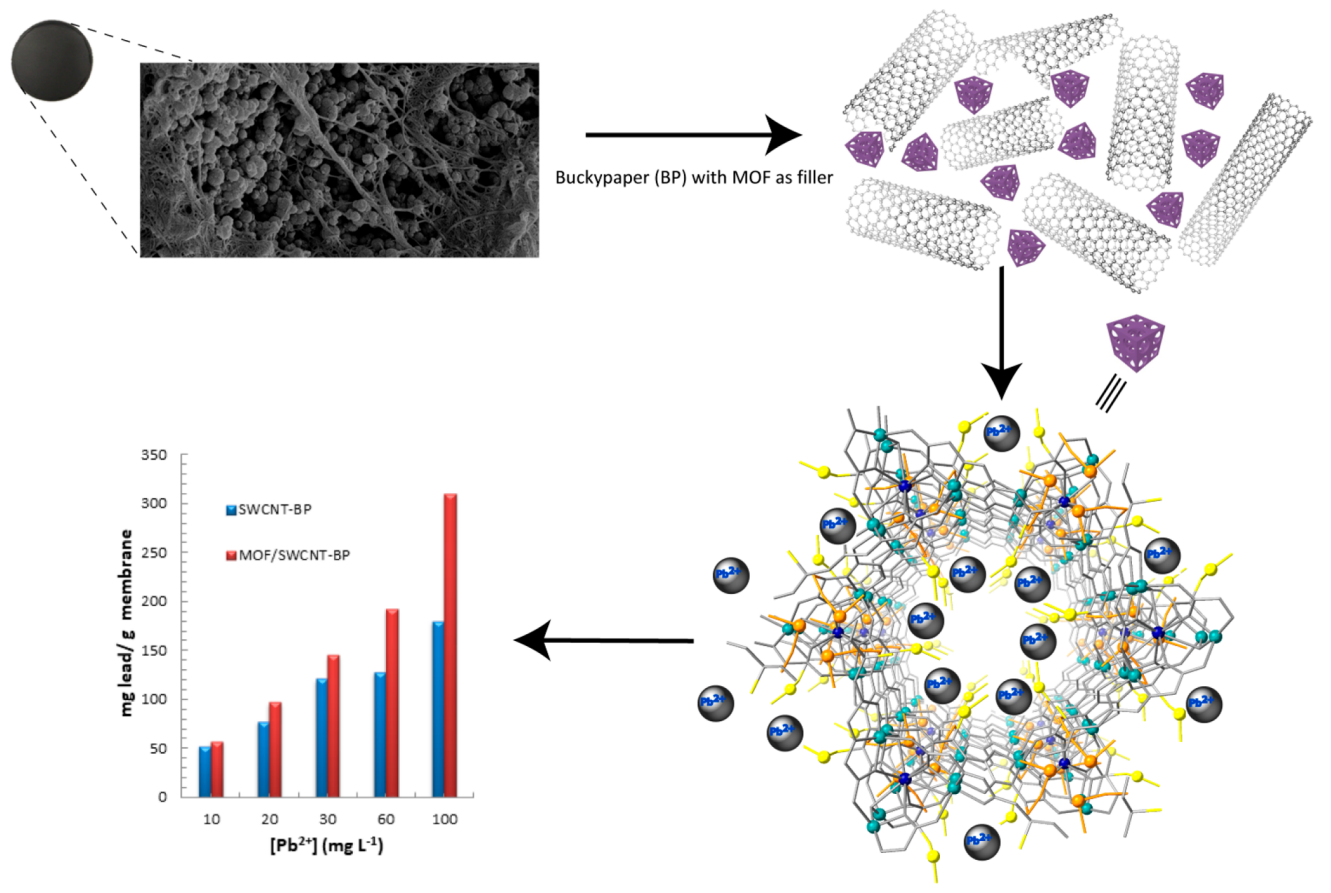
Graphical Conceptualization of the Work Here Reported Consisting of Enriching SWCNT-BP with MTV-MOF to Produce an **MTV-MOF/SWCNT-BP** Membrane The high chemical affinity of the amino acid residues of methylcysteine and methionine is exploited to recover selectively Pb^2+^ in both low and high ion concentration regimes, even in the presence of interfering ions, with an increase in adsorption capacity up to 42% with respect to neat SWCNT-BP.

Having in mind the above-mentioned points, and with the aim to make a step forward toward the concrete application of MOF-based technology to water remediation, we have investigated the integration of MTV-MOFs with SWCNT-BPs. In order to do so, we first focused on the development of a novel MTV-MOF, each component of which has been carefully selected on the basis of our knowledge of MTV-MOFs for water remediation. Remarkably, this novel material allowed us to perform an in-depth experimental study through SCXRD of MTV-MOF···Pb^2+^ interactions, which represent the first reported example of such host–guest interaction. Then, we developed an innovative and self-standing membrane, integrating single-walled carbon nanotube buckypaper and prepared multivariate MOF (**MTV-MOF/SWCNT-BP**), as a novel and well-performing material for lead removal from aqueous environments (Scheme 1). We have also compared the lead removal performance of the neat SWCNT-BP with **MTV-MOF/SWCNT-BP**, where we have found for the latter a slight increase in adsorption kinetics and a considerable improvement in terms of both adsorption capacity and selectivity over a wide range of lead concentrations (from 200 to 10000 ppb), even in the presence of background interfering ions. In particular, **MTV-MOF/SWCNT-BP** exhibited an improvement of 42% in adsorption capacity with respect the neat SWCNT-BP and was able to reduce the [Pb^2+^] from the dangerous 1000 ppb level to acceptable limits for drinking water, below 10 ppb, as the new lead rule from the EPA recently established. These features, together with the nice reusability, good chemical stability, ease of fabrication, and scalability, situate **MTV-MOF/SWCNT-BP** as an appealing technology for large-scale water and wastewater treatment.

## Results and Discussion

### Material Preparation and Characterization

We report the preparation of a novel MTV-MOF, obtained from the combination of the metalloligands used in two previously reported MOFs, using oxamidato ligands derived from amino acids,^43,55−63^ with formulas (Me_4_N)_2_{Cu_2_[(S,S)-methox](OH)_2_}·4H_2_O and (Me_4_N)_2_{Cu_2_[(S,S)-Mecysmox](OH)_2_}·5H_2_O, where methox and Mecysmox ligands are bis[(S)-methionine]oxalyl diamide and bis[(S)-methylcysteine]oxalyl diamide, respectively. The novel resulting MTV-MOF, prepared by using equimolar amounts of both metalloligands, possess the formula {Ca^II^Cu^II^_6_[(S,S)-methox]_1.5_[(S,S)-Mecysmox]_1.50_(OH)_2_(H_2_O)}·38H_2_O) (**1**). Single-crystal XRD measurements^64,65^ on **1** confirm its isostructurality with a previously reported MTV-MOF, {Sr^II^Cu^II^_6_[(S,S)-methox]_1.5_[(S,S)-Mecysmox]_1.50_(OH)_2_(H_2_O)}·36H_2_O^50^ (crystallographic details are provided in the Supporting Information, Table S1 and Figure S1). The MTV-MOF features hexagonal functional channels decorated with two types of thio-ether groups from the amino acid residues, being either −CH_2_CH_2_SCH_3_ or −CH_2_SCH_3_, pointing toward the accessible void spaces, which provides these materials with an excellent task-specific functional environment to sequestrate Pb^2+^ ions into the pores, and they consequently have the potential to enhance the capture properties of the hybrid membrane with respect the neat SWCNT-BP, after the preparation of the hybrid material **MTV-MOF/SWCNT-BP**. Indeed, the high affinity of sulfur toward inorganic pollutants such as Pb^2+^ toxic metal ions is a well-known phenomenon, which makes **1** a promising candidate for the preparation of composite materials—with improved mechanical properties—for metal contaminant removal from water. This approach has been followed before by embedding MOFs in mixed matrix membranes (MMM-MOFs) and evaluating the efficiency for the capture of mercury species.^42^ However, the enhanced mechanical properties of **SWCNT-BP** compared to MMMs, provided that the capture properties are maintained, makes this study worthwhile.

The gram-scale preparation of **1** follows a previously reported experimental procedure (see Experimental Section in Supporting Information).^50^ The experimental PXRD pattern of **1** is identical to the calculated one (Figure S2), which confirms the homogeneity of the bulk sample and its isostructurality to the single-crystals selected for SCXRD (see below). As a preliminary step to the preparation of **MTV-MOF/SWCNT-BP**, we evaluated the behavior of MTV-MOF **1** in the removal of Pb^2+^ from water (see the Supporting Information for details on preparation of **1**). To this end, 50 mg of a polycrystalline sample of **1** was soaked in an aqueous solution of Pb(NO_3_)_2_ (1 ppm, 10 mL) (see Table S2 and Experimental Section). Overall, **1** was capable of capturing, very efficiently, Pb^2+^ cations from the contaminated solution, as ICP-MS analyses indicate (Table S2). Thus, **1** is capable of reducing [Pb^2+^] from 1 ppm to less than 5 ppb, sufficiently close to acceptable limits for drinking water. Moreover, PXRD experiments confirm that **1** retains its crystallinity after these capture experiments (Figure S2c), and X-ray photoelectron spectroscopy (XPS) of **1** before and after the capture experiment (Figure S3) indicates that the sulfur oxidation state is not affected by Pb^2+^ loading, as it is observed from the analysis of the S 2p peak. Finally, the N_2_ adsorption isotherms at 77 K, for **1**, before and after lead capture are shown in Figure S4. Both isotherms are characteristic of microporous materials, with estimated Brunauer–Emmett–Teller (BET) surface areas of 605 (as synthesized) and 449 m^2^ g^–1^ (after capture experiments). As expected, after the lead capture we observed a reduction of N_2_ uptake. Nevertheless, **1** still presents a sizable porosity.

**Figure 1:**
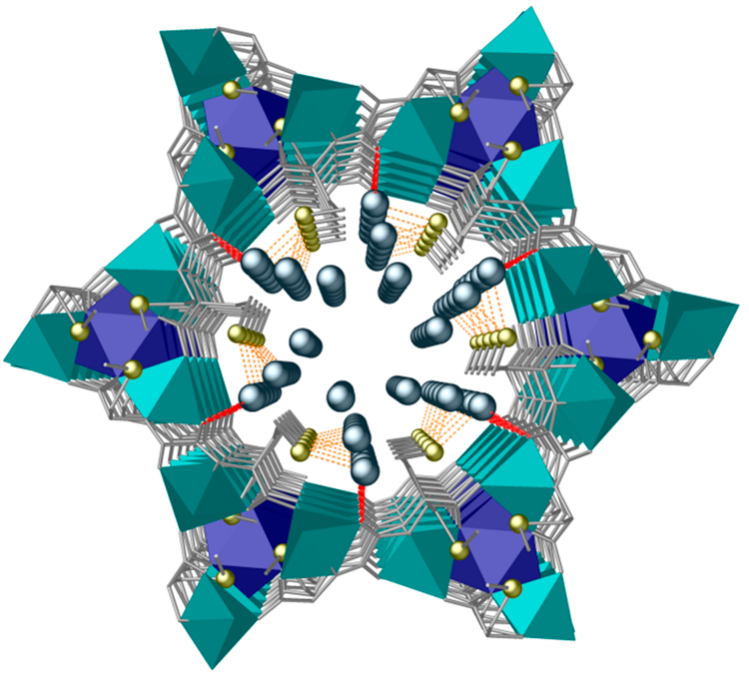
Perspective view down the *c* crystallographic axis of a single channel of **Pb@1** crystal structure determined by SCXRD, showing Pb^2+^ ions captured in pores by sulfur atoms from methionine residues through a supramolecular recognition process. S···Pb^2+^ and O···Pb^2+^ interactions are depicted by orange and red dashed lines, respectively. Color code: copper and calcium atoms from the network are represented by cyan and blue polyhedra, respectively, whereas organic ligands are depicted as gray sticks. Yellow and light blue spheres represent S and Pb atoms, respectively.

The Pb^2+^ capture was also followed and unambiguously unveiled by single-crystal X-ray diffraction (SCXRD) (see the Supporting Information and Table S1). The crystal structure of **Pb@1**, obtained by soaking crystals of **1** in saturated aqueous solutions of Pb^2+^, clearly shows Pb^2+^ metal ions residing (with both statistical and large thermal disorder) within the pores of the MTV-MOF, grasped by S···Pb^2+^ linkage [S···Pb^2+^ of 2.7(1) and 2.9(1) Å] (Figures 1, S5, and S6). An auxiliary interaction of Pb^2+^ with oxygen atoms belonging to oxamidate ligands from the MTV-MOF was also revealed, likely contributing to stabilizing the metal ions in the confined spaces [O···Pb of 2.54(5) Å] (Figure S6b,c). SCXRD data also indicates some clues regarding the Pb^2+^ capture mechanism. It clearly shows that while methionine arms act as scavengers for lead, shorter methylcysteine ones reach their stable conformation upon being confined within the most hindered voids of the crystal structure (Figure S6b). The length of the amino acid residue seems to play a key role in the process of the metal species capture. Hence, the larger length of the ethylmethyl thioether chains decorating the channels imparts more flexibility, allowing a faster approach of the target species.

On the other side, the statistical disorder exhibited by captured Pb^2+^ metal ions can be visualized as a series of snapshots, underpinning the inclusion and somewhat the transport mechanisms behind the capture within nanoconfined spaces. Indeed, the heavy metal ion is detected residing either far or close to the methionine moieties, suggesting a pre- and postrecognition from the amino acid derivative (see Figure S6 and crystallographic details in the Supporting Information). Most likely, the split on two sites with similar occupancy factors is produced by superimposed snapshots of the dynamic process within the porous crystal. It must be clarified that this disorder, as normally occurs for general statistical disorder in a crystal structure, is given by the spatial views averaged in the crystal through only one unit cell.

**Figure 2:**
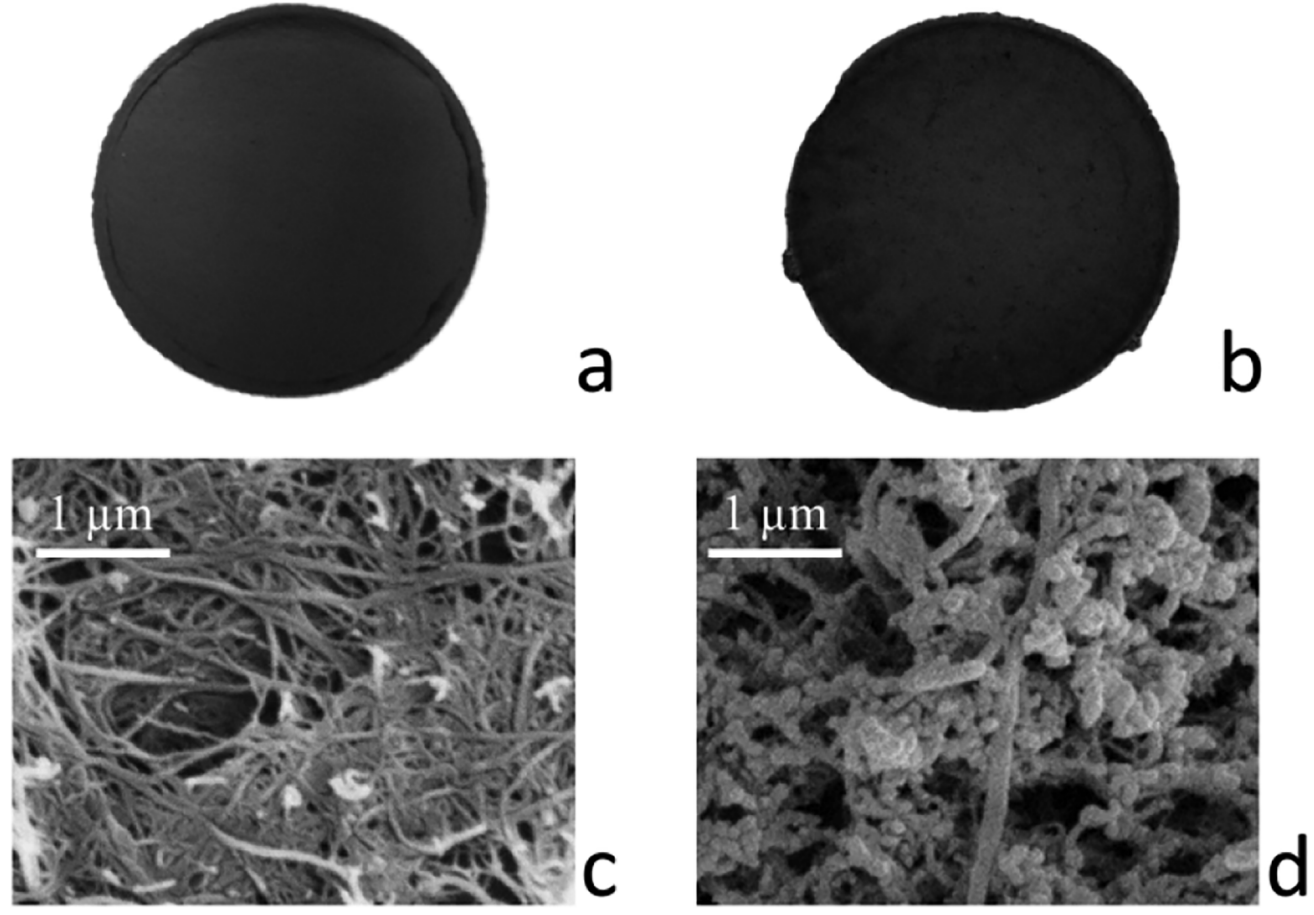
Final appearance of (a) neat SWCNT-BP and (b) **MTV-MOF/SWCNT-BP**. The average diameter of membranes was 38 ± 1 mm. SEM images of (c) SWCNT-BP and (d) **MTV-MOF/SWCNT-BP** unveiling MTV-MOF micrometer particles as aggregates of smaller nanosized primary particles.

A polycrystalline sample of **1**, with average particle dimensions of 0.1 μm, was immobilized within the porous network of entangled SWCNT-BP, thus obtaining a stable self-standing adsorbing membrane filter (Figure 2). A powder of **1** was dispersed in an optimized SWCNT-BP water solution using an ultrasonic bath. The as-made solution was filtered through the PTFE disks with a vacuum pump and then washed with ethanol. The novel membrane **MTV-MOF/SWCNT-BP** has been produced by drying the washed composite at room temperature. The detailed procedure for the composite preparation is given in the Supporting Information. Compared to the conventional method for preparing MMM-MOFs, a low cost and simple technique was employed for preparing these **MTV-MOF/SWCNT-BP** membranes, which exhibit relatively high fluxes, porosity, hydro-stability, and mechanical strength. This preparative procedure requires low amounts of eco-friendly solvents (water and ethanol) and no postsynthetic treatment, thus resulting in a more environmentally friendly process.

The SWCNT-BP selected as porous support for MTV-MOF immobilization was not an simply a bystander; it is partially functionalized with carboxylate groups (∼3,5%), which can actively and synergically cooperate in the heavy metal sequestration. Nevertheless, the mechanisms underpinning metal ion sorption within carbon nanotubes (CNTs) are very intricate, and they can be explained by the combination of several factors, such as electrostatic attraction, sorption–precipitation, and chemical interaction between the metal ions and the functional groups eventually present on the CNT surface.^66^

The optimum MOF content of 25 wt % in the SWCNT-BP was established by considering both factors: maximum lead uptake/efficiency and mechanical stability of the final membrane. Thermogravimetric analysis (TGA) evaluated the thermal stability of **MTV-MOF/SWCNT-BP**. TGA shows that the presence of MTV-MOF **1** in the hybrid membrane does not significantly influence the shape of the neat buckypaper decomposition (Figure S7). The slight changes observed are likely related to the porosity of the MOF and its water content and were observed in the temperature range of 50–250 °C for the composite membrane of **MTV-MOF/SWCNT-BP**. A mass loss of ∼47% is observed above 450 °C for both **MTV-MOF/SWCNT-BP** and neat SWCNT-BP membrane, accounting for the partial decomposition of BP.

Panels a and b of Figure 2 show neat SWCNT-BP and **MTV-MOF/SWCNT-BP** membranes, respectively, prepared under the above-mentioned optimized conditions. These membranes arranged in a circular pattern exhibit an average thickness of 60 ± 1 μm plus an average diameter of 38 ± 1 mm with a resulting average mass of the disks of 40 ± 2 mg. Density and porosity values for membranes are similar, as expected (0.60 ± 0.03 g cm^–3^ and 70 ± 5%, respectively). The pore distribution (%) for SWCNT-BP and **MTV-MOF/SWCNT-BP** membranes has been evaluated. Figure S8 shows a similar pore distribution for SWCNT-BP and **MTV-MOF/SWCNT-BP** membranes, typical of microporous materials; the estimated Brunauer–Emmett–Teller (BET) surface area for the used SWCNTs is 520 m^2^ g^–1^ (from Sigma-Aldrich). Thus, both membranes are potentially good absorbers, with pore diameters, as expected, only slightly reduced after MTV-MOF immobilization in SWCNT-BP.

The morphology of SWCNT-BP and **MTV-MOF/SWCNT-BP** membranes was evaluated through scanning electron microscopy. A typical microscopic texture of a SWCNT-BP is shown in Figure 2c, where bundles and clusters of SWCNTs are evident and likely induced by π–π and van der Waals interactions. Small spherical aggregates appear after the addition of the MTV-MOF in SWCNT-BP (Figure 2d) featuring an average diameter of roughly 0.1 μm. The stable and porous structure of SWCNT-BPs supports and stabilizes the nanosized MOF particles. Indeed, after capture experiment, no leakage was observed. Thus, the final composite guarantees good permeability together with large active surface area for the adsorption of lead from water solution.

In order to characterize the hydrophilic/hydrophobic surface properties of SWCNT-BP and **MTV-MOF/SWCNT-BP** membranes, the static contact angle has been measured for both materials. The top surfaces of both membranes feature a hydrophilic character. However, it is decreased by the presence of the MTV-MOF, with average contact-angle values of 47.5° ± 0.5° and 78.5° ± 0.5° for SWCNT-BP and **MTV-MOF/SWCNT-BP**, respectively (Figure S9a,b), which is required for water treatment membranes.^67^ These results suggest a lower hydrophilic nature for the **MTV-MOF/SWCNT-BP** compared to the neat SWCNTs. In terms of transport mechanisms, it can be seen as an added value of the chosen hybrid support.

PXRD experiments of SWCNT-BP and **MTV-MOF/SWCNT-BP** before and after capture and regeneration process have been also performed (Figure S10). They confirm the integrity of the membranes, showing the common peaks typical of SWCNTs and that the open-framework structure of the filter remains unchanged after the capture of the Pb^2+^ ion and regeneration process (*vide infra*) of **MTV-MOF/SWCNT-BP** (Figure S10).

### Capture Properties and Pb^II^ Adsorption Performance

The capture properties of **MTV-MOF/SWCNT-BP** were then evaluated through adsorption experiments and compared with those of the neat SWCNT-BP membrane. Both membrane disks were activated first by immersion in ethanol followed by heating at 80 °C under reduced pressure for 24 h prior to the sorption measurements.

**Figure 3:**
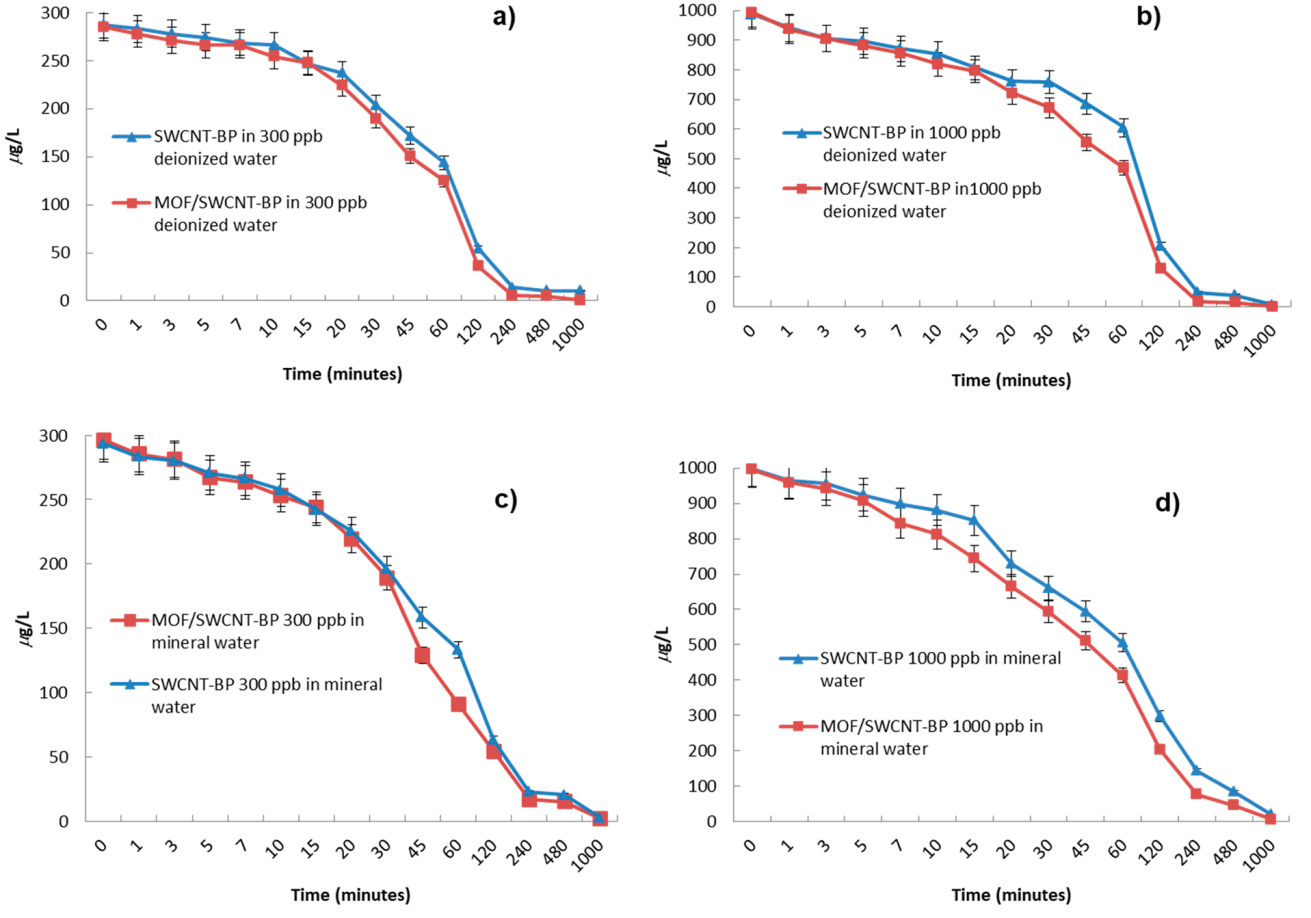
Pb^2+^ capture by neat SWCNT-BP and **MTV-MOF/SWCNT-BP** disks (diameter 38 ± 1 mm) after soaking in 200 mL of aqueous solution with [Pb^2+^] of 300 and 1000 ppb of deionized (a and b) or mineral water (c and d) in the 0–16 h interval (data from Tables S3–S8). The Pb^2+^ adsorption performance was tested on Pb(NO_3_)_2_ water solutions at room temperature under static conditions. The solid lines in the plots are a guide for the eye.

**Figure 4:**
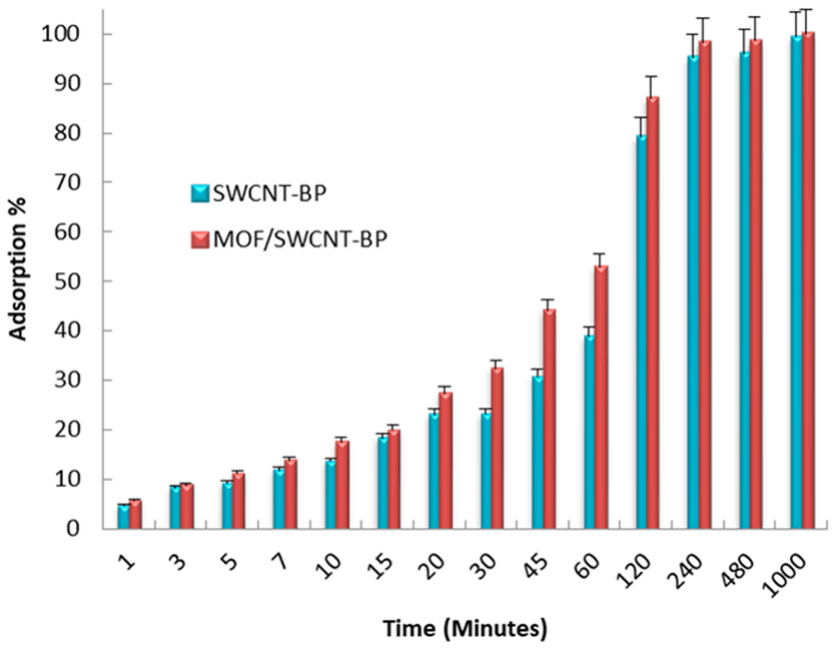
Pb^2+^ capture% by neat SWCNT-BP and **MTV-MOF/SWCNT-BP** disks (diameter 38 ± 1 mm) during soaking in aqueous solutions of 1000 ppb [Pb^2+^] [volume 200 mL of Pb(NO_3_)_2_ aqueous solution, at room temperature] in the 0–16 h interval (data from Table S4).

The initial screening was performed in batch, using distilled water and Pb(NO_3_)_2_ at different concentrations (300 and 1000 ppb). The native pH value of the solution was 6.5. At this pH, the carboxylic acid groups of SWCNT are deprotonated, but it is not supposed to affect in a remarkable way the binding performance of the MTV-MOF. The kinetic profiles and adsorption% of lead capture by SWCNT-BP or **MTV-MOF/SWCNT-BP** disk (diameter 38 ± 1 mm), after soaking them at different lead concentrations in the 0–16 h interval, are shown in Figures 3 and 4, respectively (results from the whole time range of 0–72 h are reported in Tables S3–S8).

Observing the kinetic profiles, some significant differences can be spotted regarding the capture performanc of the two membranes. The SWCNT-BP was able to reduce the lead concentration from the initial value of 300 ppb to values slightly lower than the 15 ppb action level limit in 4 h (13.7 ± 0.2 ppb), while a noteworthy improved removal capacity is observed for the **MTV-MOF/SWCNT-BP** over the same time period, with lead concentration being reduced to 5.5 ± 0.1 ppb; this is much lower than the 10 ppb trigger level and thus within acceptable limits for drinking water (Table S3). A slight increase of adsorption performance of the **MTV-MOF/SWCNT-BP** can be noticed even when the more concentrated solution was used, in line with an increase in maximum adsorption capacity at high concentrations (graphic in Scheme 1) (*vide infra*). The lead concentration was reduced from 1000 to 39.40 ± 0.4 and 14.1 ± 0.1 ppb in 8 h when the neat SWCNT-BP and **MTV-MOF/SWCNT-BP** were used, respectively (Table S3).

A doubled treatment time (16 h) is required to reduce the lead concentration under the imposed trigger level limit with the SWCNT-BP (6.65 ± 0.1 ppb) (Table S4). The obtained results clearly indicate that the presence of the MTV-MOF within the SWCNT-BP membrane improves, significantly, the removal efficiency of the neat SWCNT-BP membrane. Indeed, a considerable reduction of the lead concentration to 0.73 ± 0.02 ppb, which is under the limits required for bottled and drinking water, was observed for the same time (Table S4).

Aiming at evaluating the potential real application of these membranes, additional experiments were performed, in batch, using lead solution at different concentrations (300 and 1000 ppb) prepared with a commercial mineral water containing common and unavoidable background ions (Figure 3c,d and Tables S5–S8) at pH 6.6. The trend in capture performance is preserved, and it is evident that none of the cations present in solution–such as Na^+^, K^+^, Mg^2+^, Ca^2+^–at high concentration noticeably interfere with the adsorption of lead ions (Figures S11 and S12), even when a lower Pb^2+^ concentration of 200 ppb was used (Figure S13 and Tables S9 and S10). These results, confirming the selectivity of this material, are in line with that observed for other MOFs of the same family.^42,43,51^ Again, an improved adsorption was observed for the **MTV-MOF/SWCNT-BP** membrane at higher lead concentration. Indeed, while the adsorption ability of the SWCNT-BP and **MTV-MOF/SWCNT-BP** was comparable at 200 and 300 ppb, with lead being reduced below the established limits after 4 and 16 h, respectively, lead concentration falls from 1000 ppb to 13.9 ± 0.1 ppb in 24 h with SWCNT-BP and reached a halved value (6.9 ± 0.1 ppb) in a shorter time (16 h) when the **MTV-MOF/SWCNT-BP** was used, thus further confirming the positive effects of the MTV-MOF on the hybrid membrane performance (Tables S7 and S8).

In addition, the positive effects of the presence of MTV-MOF within SWCNT-BP are evident on the adsorption capacity and selectivity (see below) at high Pb^2+^ concentration.

Kinetic experiments evidence that the rate equation for Pb^2+^ capture in solutions follows the Lagergren first-order equation (capture experiments section, Supporting Information)
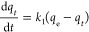
1where *q*_e_ and *k*_1_ are the lead adsorption capacity per unit of adsorbent mass (mg g^–1^) at equilibrium and the Lagergren adsorption rate constant (min^–1^), respectively (Table S11). The capture performance of **SWCNT-BPs** was improved by the incorporation of **MTV-MOF** in **SWCNT-BPs**, increasing the constant rate values for the concentrations of 300 and 1000 ppb from 0.0120 ± 0.0005 and 0.0126 ± 0.0008 min^–1^ to 0.0138 ± 0.0006 and 0.0143 ± 0.0008 min^–1^, respectively.

In terms of capacity, **MTV-MOF/SWCNT-BP** reaches a value up to 310 mg g^–1^ when soaked in an aqueous solution with [Pb^2+^] of 100 mg L^–1^. This quantity, although it could be considered modest if compared to that shown by other bulk MOFs,^32,33,39^ is quite remarkable considering that we are dealing with a membrane containing 25% of the MOF. Moreover, it results in a 42% improvement with respect to the neat SWCNT-BP, for which, in the same conditions, a maximum adsorption of 180 mg g^–1^ was observed (bar chart in Scheme 1 and Tables S12–S17). All these results undoubtedly validate that CNTs are good supports to immobilize and prevent leaching of MOFs in target solutions, a drawback often present in MOF-based mixed polymeric membranes.

**Figure 5:**
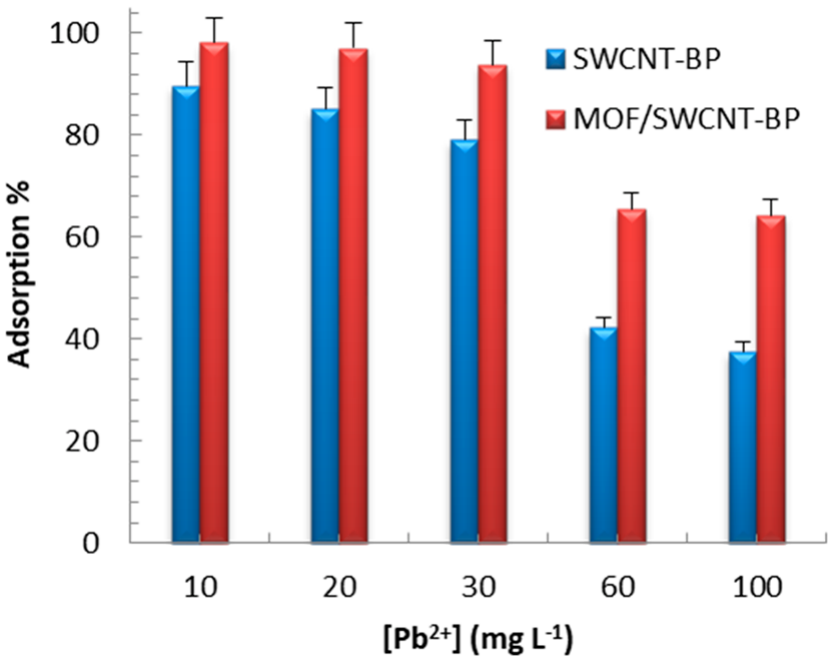
Dependence of Pb^2+^ capture% as a function of concentration by neat SWCNT-BP and **MTV-MOF/SWCNT-BP** disks in 10–100 ppm range of [Pb^2+^] [volume of 200 mL of Pb(NO_3_)_2_ aqueous solution, at room temperature for 72 h] (data from Table S12).

Finally, in order to ensure the applicability of **MTV-MOF/SWCNT-BP** against any accidental spills, similar to what occurred in Flint, we performed further adsorbing tests by using Pb^2+^ solutions at higher initial concentrations (10000, 20000, 30000, 60000, and 100000 ppb) (Figure 5 and Tables S13–S17). After 72 h of treatment, a removal efficiency up to 90% was observed for the SWCNT-BP membrane at 10000 ppb, with final Pb^2+^ concentration in water of ca. 1200 ppb. With respect to this value, a further reduction of the final concentration of 82% was obtained with the **MTV-MOF/SWCNT-BP** membrane in the same conditions, with the final lead concentration being slightly higher than 200 ppb, corresponding to a removal efficiency of 98% (Table S12). When the initial lead concentration rises further, the removal efficiency of both membranes decreases, with **MTV-MOF/SWCNT-BP** still assuring the better performance: after 72 h of treatment the removal efficiency values were 79 and 94% for initial lead concentration of 30000 ppb (Table S15) and 42 and 66% for initial lead concentration of 60000 ppb (Table S16) for SWCNT-BP and **MTV-MOF/SWCNT-BP**, respectively.

### Selectivity toward Common Interfering Metal Ions Al^3+^ and Fe^3+^

The already described improved lead uptake efficiency of **MTV-MOF/SWCNT-BP** compared to neat SWCNT-BP is also accompanied by a remarkable increase in selectivity. In particular, we evaluated the lead capture performance of both **MTV-MOF/SWCNT-BP** and SWCNT-BP membranes toward Pb^2+^ in the presence of other interfering metal cations, usually found in drinking water, such as Al^3+^ and Fe^3+^ cations. In order to do so, both **MTV-MOF/SWCNT-BP** and SWCNT-BP were soaked in aqueous solutions containing an initial concentration of 1000 ppb of each metal, where we observed **MTV-MOF/SWCNT-BP** showed higher selectivity toward Pb^2+^ cations (Tables S18 and S19).

Thus, for **MTV-MOF/SWCNT-BP** a removal efficiency of 97.8% for lead can be observed, which corresponds to a final lead concentration of 22 ± 1 ppb, whereas the corresponding removal efficiency for the SWCNT-BP was only 90% for Pb(II) (99 ppb of lead remaining in solution after treatment) (Tables S18 and S19).

The calculation of lead distribution coefficients, *K*_D_, between membranes and solutions both in neat and multicomponent (Pb, Fe, and Al) solutions (see Table S20) confirmed the strong affinity between Pb^2+^ ions and membranes. Even if a decrease in *K*_D_ values was observed for increasing lead concentrations and in the presence of competitive ions, it is worth noting that the incorporation of **MTV-MOFs** in **SWCNTs** increased the ion affinity in all cases reported in Table S20.

Overall, all these capture results indicate that **MTV-MOF/SWCNT-BP** is capable of reducing [Pb^2+^] in contaminated solutions to acceptable limits for drinking water. These results situate **MTV-MOF/SWCNT-BP** among the most efficient materials for lead decontamination and as the most efficient MOF-based material for this purpose.^28−40^

**Figure 6:**
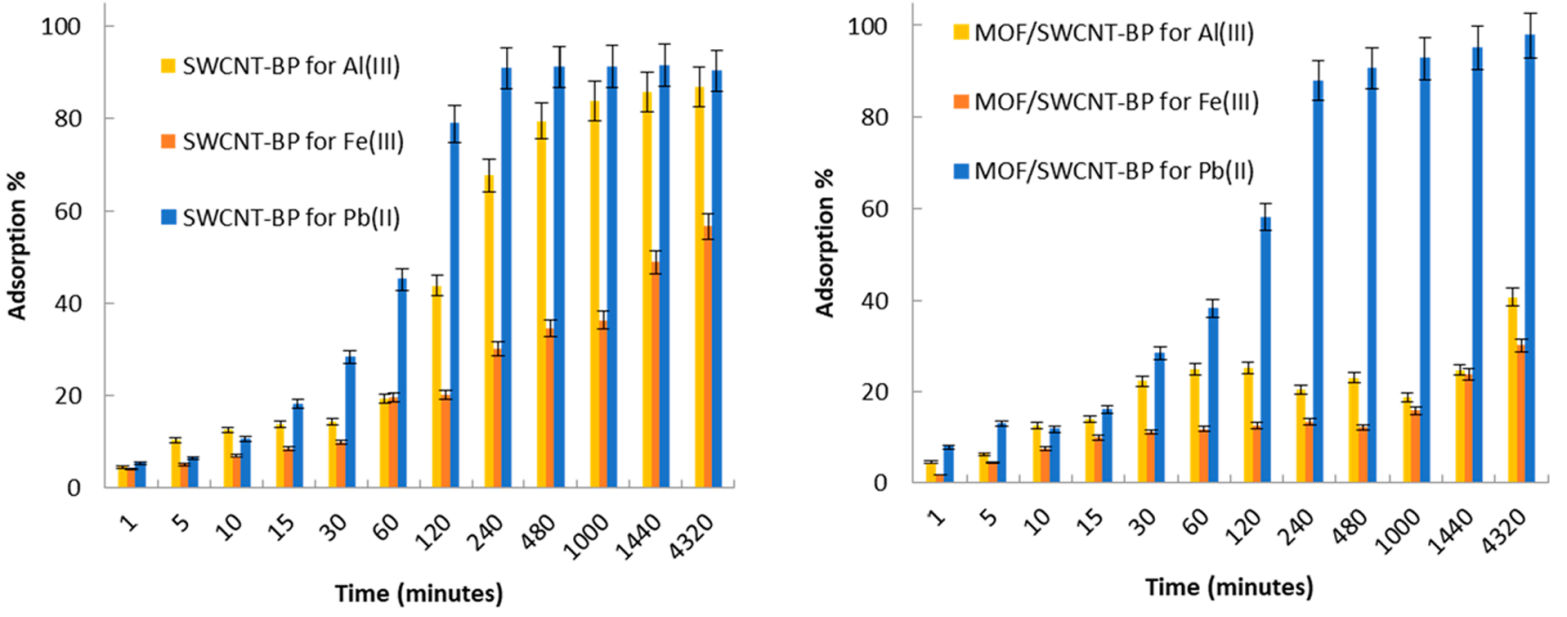
Selectivity for cation adsorption in 1000 ppb [Pb^2+^], [Fe^3+^], and [Al^3+^] solutions by **MTV-MOF/SWCNT-BP** and SWCNT-BP membranes soaked in a volume of 200 mL, in the 0–72 h interval (data from Tables S18 and S19). Membrane disks were placed in 1 ppm Pb(NO_3_)_2_, FeCl_3_ and AlCl_3_ aqueous solution at room temperature (25 °C). To avoid precipitation of Fe^3+^ and Al^3+^ as metal hydroxides from the aqueous solution during selectivity experiment, the addition of nitric acid for the stabilization has been performed until pH 2.

As stated above, a greatly increased absorption ability for Al(III) and Fe(III) was observed only for the SWCNT-BP membrane (removal efficiency of 87 and 57% for Al(III) and Fe(III), respectively), when compared to **MTV-MOF/SWCNT-BP** (removal efficiency of 41 and 30% for Al(III) and Fe(III), respectively). These results are shown in Figure 6 and clearly demonstrate an outstanding selectivity of **MTV-MOF/SWCNT-BP** toward Pb^2+^ (Figure 6b), which decreases, significantly, in neat SWCNT-BP (Figure 6a). Overall, these results further confirm that the introduction of the MTV-MOF brings added value to SWCNT-BP, thus facilitating its real implementation.

These trends are maintained at higher concentrations. When using solutions with an initial concentration of 10000 ppb for each metal (Figure S14 and Tables S21 and S22), **MTV-MOF/SWCNT-BP** still displays higher selectivity and removal efficiency for Pb(II) ions when compared with SWCNT-BP, being as high as 97% for lead for the composite membrane and 64% for the buckypaper. This behavior should be attributed to the high chemical affinity of thioether functions for Pb^2+^ soft metal ions. Methionine residues, pointing within pores of the MTV-MOF, capture Pb^2+^ ions as unveiled by the SCXRD study (Figure 1). These thioether-functionalities, and consequently holding mechanisms, are not present in carbon nanotubes. In neat SWCNT-BP, there exists an oxygen-rich environment related, as above-mentioned, to partial functionalization with carboxylate groups (∼3.5%); this and physisorption mechanisms are likely the main actors in the capture processes. These nonspecific interactions are most likely at the origin of the greater affinity unveiled for Al^3+^ and Fe^3+^ in SWCNT-BP (Figure 6). Further evidence of the synergistic effect of MTV-MOF and SWCNT-BP in the hybrid membrane is supported by SEM-EDX analysis on both membranes after Pb^2+^ capture (Figure S15). Images confirm a still homogeneous distribution of MTV-MOF in SWCNT-BP and a homogeneous adsorption in both membranes of Pb(II).

### Perspective on Industrial Feasibility and Scale Up: Mechanical Analysis and Regeneration Process

In order to ensure that the excellent mechanical properties of BP are maintained when preparing composites, the mechanical properties of **MTV-MOF/SWCNT-BP** were evaluated and compared to those shown by pure SWCNT-BP. The presence of MTV-MOF in SWCNT-BP causes a slight decrease in the Young’s modulus from 1.65 ± 0.03 GPa (neat SWCNT-BP) to 1.54 ± 0.02 GPa (**MTV-MOF/SWCNT-BP**). Nevertheless, these measurements confirm the high mechanical stability of both membranes (see Figure S16).^68^

From the perspective of potential industrial applications, and being aware of the known stability for either SWCNT-BP^68^ or MTV-MOF^49^ after regeneration processes, the stability of the **MTV-MOF/SWCNT-BP** membrane after a regeneration process was also evaluated. In this regard, recovered Pb^2+^ was extracted after suspension of the membrane in a 10% (v/v%) aqueous solution of 2-mercaptoethanol for 24 h. The reusability of **MTV-MOF/SWCNT-BP** after the extraction process was studied over five subsequent cycles of adsorption and regeneration. The results demonstrate the stability, confirmed by PXRD measurements (Figure S10), and still good efficiency of the hybrid membrane (Table S23), which recovers up to 85.1% of the original adsorption capacity, thus confirming the suitability of **MTV-MOF/SWCNT-BP** for industrial applications.

According to our protocol, we asked a company (potentially involved in the industrial feasibility and scale up) to make a first estimation of the price of the MTV-MOF at a 5–15 kg scale. At 5 kg scale using our procedure they estimate a production cost of 8000 €/kg (including tool depreciation, labor cost, raw materials, and energy) that could be reduced to <4000 €/kg for 15 kg scale by having a more industrial sourcing of methionine and methylcysteine amino acids. As far as BP is concerned, we have an estimation of 1000 €/kg. Of course, the main fraction of the cost comes from the MOF and reactants, but considering that membranes are prepared with a MOF content of 25 wt % in the SWCN-BP, it could be considered cheaper with respect to pure powder of MOF-based technologies.

It is more difficult at this stage to estimate the operating cost of the Pb^2+^ removal (in €/g of Pb^2+^) and to compare this cost with the expectation of the market or other existing technologies. However, we think that the balance between the membrane production, cost of the regeneration/recovery steps, and their low environmental impact is the key point.

## Conclusions

In summary, we have reported the synthesis, characterization, and lead removal efficiency of a novel material made up of a single-walled carbon nanotube buckypaper incorporating a novel multivariate MOF (**MTV-MOF/SWCNT-BP**). The combination of SWCNT-BP and MTV-MOF, materials that exhibit great capture properties individually, originates a biocompatible, highly stable adsorbent membrane—**MTV-MOF/SWCNT-BP**—with high performance for Pb(II) removal from aqueous solution. It represents a step forward with respect to the previous composite we reported so far,^68^ where the MOF embedded inside the CNTs was completely different. In that case, it was prepared with a different amino acid derivative, threonine, owning an alcoholic chemical functionality of the type −CH(CH_3_)OH confined within the pores of the MOF and exhibiting a consequent chemical affinity for rare earth elements (REEs) as target metal ions. In the present work we immobilized a multivariate-MOF, which is a new subclass of MOF, exhibiting multiple chemical functionalities. Here, we have used two chemical amino acid derivatives from methionine and methylcysteine, giving a final MTV-MOF with both −CH_2_SCH_3_ and −CH_2_CH_2_SCH_3_ functionalities confined within the pores of the MOF and exhibiting a totally different binding affinity when compared with the threonine one. Furthermore, the characterizations of as-prepared composite membranes indicated that the MTV-MOF particles are stably enmeshed into the BP skeleton.

Both the SWCNT-BP and the **MTV-MOF/SWCNT-BP** featured appreciable values of maximum adsorption capacities. However, **MTV-MOF/SWCNT-BP** displayed a remarkable increase of up to 42%, which is the only one exhibiting a remarkable selectivity for Pb(II) ions, even in highly concentrated multicomponent solutions, over a wide range of lead concentrations. These outstanding properties are likely related to MTV-MOF host–guest interactions, unequivocally unveiled by SCXRD measurements. For Pb(II) solution concentration in the range of 200–1000 ppb, the lead content was reduced well below the current established EPA and WHO trigger level and within the drinkable regime (<10 ppb), with the **MTV-MOF/SWCNT-BP** membrane assuring a faster and more efficacious decrease of the lead level in water. When the solution concentration was further increased (10000–50000 ppb), the **MTV-MOF/SWCNT-BP** membrane demonstrated superior removal efficiency with respect to the pristine SWCNT-BP, guaranteeing lead removal higher than 90% for initial lead concentration up to 30000 ppb. To further increase the removal efficiency and reduce the time of treatment, multiple filtration units could be readily connected in series to achieve higher performance and consequently reduce the water concentration under the required limit. The outstanding performance of the composite membrane is retained even in the presence of competitive ions, such as iron(III) and aluminum(III), with removal efficiency up to 97% for lead when solutions containing 10000 ppb of each metal ions were treated, in contrast to what is observed for the neat membrane. Furthermore, the structural stability of the **MTV-MOF/SWCNT-BP** was maintained upon lead adsorption/desorption cycles, and complete regeneration was achieved for up to five cycles. **MTV-MOF/SWCNT-BP** has great potential in the field of water treatment and can be effectively used as a reliable adsorbent for Pb(II) removal for household drinking water, as well as in industrial treatment plants for water and wastewater decontamination.

## Supporting Information Available

Experimental preparation, physical and analytical characterization, and crystallographic refinement details (Tables S1–S20 and Figures S1–S11) (PDF)
CCDC 2128259 (CIF)
CCDC 2128260 (CIF)

## Supplementary Material

an2c00280_si_001.pdf

an2c00280_si_002.cif

an2c00280_si_003.cif
