# Supplementary material for: Multivariate Metal–Organic Framework/Single-Walled Carbon Nanotube Buckypaper for Selective Lead Decontamination
Source: ACS Appl Nano Mater. 2022 Apr 1;5(4):5223–33. doi: 10.1021/acsanm.2c00280 (PMC9039961; doi:10.1021/acsanm.2c00280)
Supplement: Supplementary file 1 — an2c00280_si_001.pdf [file an2c00280_si_001.pdf]

Supporting Information (SI) for the manuscript:

Multivariate Metal-Organic Framework/Single-Walled Carbon Nanotube Buckypaper for Selective Lead Decontamination

Mariafrancesca Baratta,[†,§] Teresa Fina Mastropietro,[†,§] Rosaria Bruno,[†,§] Antonio Tursi,[†,§] Cristina Negro,[‡] Jesús Ferrando-Soria,*[,‡] Alexander I. Mashin,[◊] Aleksey Nezhdanov,[◊] Fiore P. Nicoletta,*[,#] Giovanni De Filpo,*[,†] Emilio Pardo*[,‡] and Donatella Armentano*[,†]

[†] Dipartimento di Chimica e Tecnologie Chimiche (CTC), Università della Calabria, Rende 87036, Cosenza, Italy

[‡] Instituto de Ciencia Molecular (ICMol), Universidad de Valencia, 46980 Paterna, Valencia, Spain

[◊] Applied Physics & Microelectronics, Lobachevsky State University of Nizhni Novgorod, Nizhni Novgorod, Russian Federation

[#] Dipartimento di Farmacia e Scienze della Salute e della Nutrizione, Università della Calabria, 87036, Rende, Italy

[§]These authors have equally contributed to this work

*E-mail: jesus.ferrando@uv.es; giovanni.defilpo@unical.it; emilio.pardo@uv.es; donatella.armentano@unical.it; f.nicoletta@unical.it

Experimental Section

Chemicals:

All chemicals were of reagent grade quality. They were purchased from commercial sources and used as received. SWCNTs (characterized by an average diameter of 1.4 ± 0.1 nm and an average length longer than 5 μm) and carboxylic acid functionalized SWNTs (COOH-SWCNTs with bundle average dimensions ranging from 4 to 5 nm in diameter and from 0.5 to 1.5 μm in length, as reported in the datasheet from Sigma-Aldrich, Milan, Italy) were used for the preparation of SWCNT solutions.

Compound $\{Ca^{II}Cu^{II}_6[(S,S)\text{-methox}]_{1.5}[(S,S)\text{-Mecysmox}]_{1.50}(OH)_2(H_2O)\}\cdot 38H_2O$ (1) (**MTV-MOF**) was prepared as reported earlier for similar MTV-MOF containing Sr(II).[65]

Synthesis of $\{Ca^{II}Cu^{II}_6[(S,S)\text{-methox}]_{1.50}[(S,S)\text{-Mecysmox}]_{1.50}(OH)_2(H_2O)\}\cdot 38H_2O$ *(**1**):*

Well-shaped hexagonal prisms of **1** suitable for SCXRD were obtained by slow diffusion in H-shaped tubes of aqueous solutions containing stoichiometric amounts of $(Me_4N)_2\{Cu_2[(S,S)\text{-methox}](OH)_2\}\cdot 4H_2O$ (0.131 g, 0.18 mmol) and $(Me_4N)_2\{Cu_2[(S,S)\text{-Mecysmox}](OH)_2\}\cdot 5H_2O$ (0.129 g, 0.18 mmol) in one arm and $CaCl_2$ (0.022 g, 0.2 mmol) in the other. They were isolated by filtration on paper and air-dried. A gram-scale procedure was also carried out successfully by mixing greater amounts of $(Me_4N)_2\{Cu_2[(S,S)\text{-methox}](OH)_2\}\cdot 4H_2O$ (4.37 g, 6 mmol) and $(Me_4N)_2\{Cu_2[(S,S)\text{-Mecysmox}](OH)_2\}\cdot 5H_2O$ (4.32 g, 6 mmol) in water (60 mL). Another aqueous solution of $CaCl_2$ (0.440 g, 4 mmol) was added dropwise to the resulting deep green solution and the final mix was allowed to react, under stirring, for 6 hours. Afterwards, the material was isolated by filtration and characterized by C, H, N, S, analyses to give a final formula of $\{Ca^{II}Cu^{II}_6[(S,S)\text{-methox}]_{1.50}[(S,S)\text{-Mecysmox}]_{1.50}(OH)_2(H_2O)\}\cdot 38H_2O$. Anal. calcd. for **1**: $C_{33}Cu_6CaS_6H_{122}N_6O_{59}$ (2161.04): C, 18.34; H, 5.69; S, 8.90; N, 3.89%. Found: C, 18.21; H, 5.57; S, 8.77; N, 3.96%; IR (KBr): ν = 1625 and 1596 cm^{-1} (C=O).

Preparation of $[Pb(NO_3)_2]_{0.5}@\{Pb^{II}Cu^{II}_6[(S,S)\text{-methox}]_{1.5}[(S,S)\text{-Mecysmox}]_{1.50}(OH)_2(H_2O)\}\cdot 7H_2O$

$[Pb(NO_3)_2]$@1.

Well-formed hexagonal green prisms of **[Pb(NO$_3$)$_2$]@1**, which were suitable for X-ray diffraction, were obtained by soaking crystals of **1** (5.0 mg) in a saturated H_2O/CH_3OH (1:1) solution of $Pb(NO_3)_2$ for 6 hours. The crystals were washed with water, isolated by filtration on paper and air-dried. **[Pb(NO$_3$)$_2$]@1:** Anal.: calcd for $C_{33}Cu_6CaH_{60}PbS_6N_8O_{34}$ (1933.76): C, 20.50; H, 3.13; S, 9.95; N, 5.79%. Found: C, 20.67; H, 3.11; S, 9.89; N, 5.90%. IR (KBr): ν = 1623 cm^{-1} (C=O). **[Pb(NO$_3$)$_2$]@1.**

Preparation of membranes:

Buckypaper membranes were obtained by filtration of solutions of SWCNT and COOH-SWCNTs. Generally, 40 mg of SWCNT mixtures were dispersed in 200 mL of a 0.4% TRITON X100 water solution by an ultrasonic bath (model M1800H-E, Bransonic, Danbury, CT, USA) for 30 min. Then, solutions were filtered through PTFE disks with a vacuum pump (pressure = −0.04 bar), washed several times with methanol and, finally, dried at room temperature. All chemicals were purchased from Sigma-Aldrich, Milan, Italy. It is well known that several preparation factors influence the final BP membrane properties, including the SWCNT solution sonication time, the vacuum depression magnitude used during the filtration of SWCNT solutions, the filter porosity and composition.[68] In addition, the main problem found in this study was the best compromise in terms of weight percentage between long SWCNTs, which ensure the gain of self-standing and flexible BP disks, and short COOH-SWCNTs, which ensure a higher lead adsorption. After several trials, the best weight ratio was SWCNTs:COOH-SWCNTs = 3:1, which gave detachable SWCNT disks from polymer filters and self-sustaining BPs (Figure 2a).

MTV-MOF/SWCNT-BPs were obtained by dispersing MOF in the optimized SWCNT solution and applying the procedure previously outlined for neat **SWCNT-BP**. The largest amount of MTV-MOF, which was possible to disperse in the optimized SWCNT solution without losing detachability and self-sustainability of the final BP disks, was 25% w/w (Figure 2b).

Both **SWCNT-BP** and **MTV-MOF/SWCNT-BP** were flexible disks with an average diameter of 38 ± 1 mm and an average thickness of 60 ± 1 μm.

Chemical-physical techniques:

Elemental (C, H, N) and powder X-ray Diffraction analyses (of the pure MOF) were performed at the Microanalytical Service of the University of Calabria. ICP-MS analyses for the Pb^{2+} and

interferent ion capture experiments on membrane (*vide infra*) were performed at the Department of Chemistry of the University of Calabria. FT–IR spectra were recorded on a Perkin-Elmer 882 spectrophotometer as KBr pellets. All characterizations confirmed the purity of the sample when compared with the previously work reported by us.[50]

X-ray Powder Diffraction Measurements:

Polycrystalline sample of **1** to test purity of the bulk, was introduced into 0.5 mm borosilicate capillaries prior to being mounted and aligned on a Bruker D2 Phaser powder diffractometer, using Cu Kα radiation (λ = 1.54056 Å). Five repeated measurements were collected at room temperature (2θ = 2–40°) and merged in a single diffractogram. A polycrystalline sample of **1** was also measured after lead capture experiments following the same procedure.

The flexible disks of an average diameter of 34 ± 1 mm for both SWCNT-BP and MTV-MOF/SWCNT-BP have been allocated on a plate sample holder, then mounted and aligned on a Bruker D2 Phaser powder diffractometer, using Cu Kα radiation (λ = 1.54056 Å). Three repeated measurements were collected at room temperature (2θ = 2–60°) and merged in a single diffractogram.

X–ray photoelectron spectroscopy (XPS) measurements:

Samples of **1**, before and after capture experiments, were prepared by sticking, without sieving, the samples onto a molybdenum plate with scotch tape film, followed by air drying. Measurements were performed on a K-Alpha™ X-ray Photoelectron Spectrometer (XPS) System using a monochromatic Al K(alpha) source (1486.6 eV). As an internal reference for the peak positions in the XPS spectra, the C1s peak has been set at 284.8 eV.

Gas adsorption:

The N_2 adsorption-desorption isotherms at 77 K, were carried out, on policrystalline samples of of **1**, before and after capture experiments, with a BELSORP-mini-X instrument. Samples were first activated with methanol and then evacuated at 348 K during 19 hours under 10^{-6} Torr prior to their analysis.

Membrane characterization:

The morphology of **SWCNT-BP** and **MTV-MOF/SWCNT-BP** was characterized by scanning electron microscopy with a Leica LEO 420 (Leica Microsystems, Cambridge, England) scanning

electron microscope with an accelerating voltage of 10 kV. Samples were covered with an ultrathin gold layer using a sputter coater.

Figures 2c and 2d show the morphology of **SWCNT-BP** and **MTV-MOF/SWCNT-BP** membranes. SWCNT-BP morphology is characterized by the typical BP microscopic texture with bundles and clusters of SWCNTs arising from π–π and van der Waals interactions. The addition of MTV-MOFs in **SWCNT-BP** is evidenced in Figure 2d by the presence of small spherical particles with an average size lower than 1 μm and formed by smaller primary particles with an average diameter of ≈ 100 nm. The highly porous structure of **SWCNT-BP**, firmly hosting the nanosized MTV-MOF particles (no leakage was observed at the end of experiments), guarantees a high permeability and a large surface area for the contact and successive adsorption of ions present in the test solutions. MTV-MOF particles are homogenously distributed inside the membrane.

Thermogravimetric analysis:

The thermogravimetric analysis was performed on **SWCNT-BP** and **MTV-MOF/SWCNT-BP** samples under a dry N_2 atmosphere with a Mettler Toledo TGA/STDA 851^e thermobalance. The experiments were carried out within a temperature range from 25 °C to 800 °C at a heating rate of 10 °C/min. Approximately 20 mg of each sample were placed in a ceramic pan for the measurement.

Capture experiments:

The Pb^{2+} adsorption performance of **SWCNT-BP** and **MTV-MOF/SWCNT-BP** were tested on $Pb(NO_3)_2$ water solutions and multielement solution of $FeCl_3$, $AlCl_3$ and $Pb(NO_3)_2$ at room temperature and under static conditions (Tables S3-S10, S12-S19, S21-S23).

SWCNT-BP and **MTV-MOF/SWCNT-BP** membrane disks were placed in beakers containing 200 mL of $Pb(NO_3)_2$ solution (of deionized and mineral water) at room temperature (25°C).

For selectivity experiments, **SWCNT-BP** and **MTV-MOF/SWCNT** membranes were placed in 100 mL beakers containing 1 and 10 ppm of $Pb(NO_3)_2$, $FeCl_3$ and $AlCl_3$ aqueous solution at room temperature (25°C). All solutions were stirred by an orbital shaker (PSU-10i, Biosan, Italy). Each experiment was performed in triplicate and results are reported as average values of ± 3SD. The stabilization of the Pb^{2+} in view of the ICP-MS analysis occurred by adding nitric acid with a purity of 98 %. The ICP-MS data are reported considering the dilution factor. For selectivity experiment, to avoid precipitation of Fe^{3+} and Al^{3+} as hydroxide from the aqueous solution, the addition of nitric acid for the stabilization has been performed until pH =2.

The Pb^{2+} adsorption performance of **SWCNT-BPs** and **MTV-MOF/SWCNT-BPs** reported in Figure 3 (data from Tables S2 and S3) have been fitted under the hypothesis that the lead adsorption capacity of adsorbent (mg g^{-1}) follows the Lagergren-first-order equation[1,2]:

$$\frac{dq_t}{dt} = k_1(q_e - q_t) \qquad (1)$$

where q_e and k_1 are the lead adsorption capacity per unit of adsorbent mass (mg g^{-1}) at equilibrium and the Lagergren adsorption rate constant (min^{-1}), respectively. After integration, the following equation holds:

$$q_t = q_e(1 - e^{-k_1 t}) \qquad (2)$$

The experimental amount of adsorbed metal ions ($q_{exp}(t)$, mg g^{-1}) can be expressed as follows:

$$q_{exp}(t) = \frac{(C_0 - C_t)}{m} V \qquad (3)$$

where C_0 and C_t are the lead concentration in the solution at time zero and t, respectively, m is the mass of the used membranes, and V is the volume of lead solution.
Experimental data were fitted as a function of time by OriginPro 2019 Software.[3] Non-linear optimization method by Origin software was used instead of linear regression in order to avoid any distortions created in the original error distribution.[4]
The obtained Lagergren adsorption rate constants, k_1 (min^{-1}), for **SWCNT-BPs** and **MTV-MOF/SWCNT-BPs** at different initial lead concentrations are reported in Table S11.
The fit of the kinetic profiles of **SWCNT-BP** and **MTV-MOF/SWCNT-BP** membranes agrees with the findings obtained from their direct observation. The capture performance of **SWCNT-BPs** was improved by the incorporation of **MTV-MOF** in **SWCNT-BPs** increasing the constant rate values for the concentrations of 300 and 1000 ppb from 0.0120 ± 0.0005 and 0.0126 ± 0.0008 min^{-1} to 0.0138 ± 0.0006 and 0.0143 ± 0.0008 min^{-1}, respectively.

Distribution coefficients calculation:

The lead distribution coefficients, K_D, between membranes and solutions both in neat and multi-component (Pb, Fe, Al) solutions were calculated, in agreement with reference,[5] according to the following equation:

$$K_D = \frac{[Pb^{2+}]_{mem}}{[Pb^{2+}]_{sol}} \qquad (4)$$

where $[Pb^{2+}]_{mem}$ (mg kg^{-1}) and $[Pb^{2+}]_{sol}$ (mg L^{-1}) are the lead ions concentrations at equilibrium adsorbed onto the membrane and present in the solution, respectively. Table S20 shows the $\log K_D$ values calculated for the lead initial concentrations of 1000 ppb and 10000 ppb in neat and multi-component (Pb, Fe, Al) solutions.

Generally a $\log K_D$ value larger than 5 is indicative for a high affinity of ions to be adsorbed onto a substrate.[6] As reported in Table S20 at Pb^{2+} concentration of 1000 ppb, the $\log K_D$ values for lead neat solutions are larger than 5 both for SWCNT-BPs and MTV-MOF/SWCNT-BPs, confirming the strong affinity between Pb^{2+} and membranes.

As expected, the $\log K_D$ values decrease for increasing lead concentrations as a consequence of the saturation of the active sites present in membranes. Similar trends, but with lower values, were found for the $\log K_D$ in multi-component solutions, confirming the competitive affinity of iron and aluminum ions for both SWCNT-BP and MTV-MOF/SWCNT-BP membranes. It is worth noting that the substitution of SWCNTs with MTV-MOFs increases the ions affinity in all cases reported in Table S20.

ICP-MS analyses:

The Pb^{2+} concentrations together with the Al^{3+}, Fe^{3+}, Na^{+}, K^{+}, Mg^{2+} tested ions were determined by utilizing an inductively coupled plasma-mass spectrometer (ICP-MS iCAP™ TQ Thermo Fisher Scientific, USA) equipped with a Peltier cooled high purity quartz baffled cyclonic spray chamber, a concentric borosilicate glass nebulizer, a wide 2.5 mm internal diameter quartz injector, a nickel sample and two skimmer cones with 1.1 mm and 0.5 mm diameter orifices, respectively. The ICP torch was a demountable single piece quartz torch. The samples were collected by a Thermo Scientific™ Autosampler Housing with a peristaltic pump equipped with three-stop flared PVC pump tubing. A multielement standard solution was used to calibrate the instrument using different analytical concentrations (0.5, 5, 10, 20, 50 and 100 ppb, R^2 of calibration curve ≈ 1 and a limit of detection, LOD, of about 0.015 ppb). Ultrapure deionized water (18.3 MΩ cm, Arioso, Human Corporation, Korea) was used for the aqueous solutions preparation after filtration by 0.45 µm filter (Millex Syringe Filter, Merck, Darmstadt, Germany). Each experiment was performed in triplicate and results are reported as average values ± 3 SD. Data reported in Tables S2-S10, S12-S19, S21-S23.

EDX measurements:

Scanning Electron Microscopy (SEM) measurements were carried out for both SWCNT-BP and BioMOF/SWCNT-BP using a LEO 420 scanning electron microscope (SEM, Zeiss) (Vacuum conditions: 8x10-6 Torr, accelerating voltage: 15 kV) coupled with an Energy Dispersive X–ray (EDX) detector. The EDX module was an INCAx–Sight Oxford Instruments (Vacuum conditions: 8×10^{-6} Torr, iProbe: 650 pA, Current: 15 kV). Samples were gold metallized by an Auto Sputter Coater (Agar). Both membranes were suspended in a 10 ppm $Pb(NO_3)_2$ aqueous solution for 24 h for EDX measurements.

Contact angle measurements:

Static contact angle values of **SWCNT-BP** and **MTV-MOF/SWCNT-BP** were measured with a goniometer (Nordtest, Serravalle Scrivia AL, Italy) at 25 °C. A drop (2 µL) of water was put onto the sample surface by a micro-syringe and measurements were carried out by setting the tangents on both visible edges of the droplet on five different positions of each sample and calculating the average value of the measurements. The hydrophilic character of **SWCNT-BP** and **MTV-MOF/SWCNT-BP** top surfaces was confirmed by contact-angle measurements, which gave average contact-angle values of 47.5° ± 0.5° and 78.5° ± 0.5°, respectively, Figures S5a and S5b.

Mechanical properties:

The mechanical properties of **SWCNT-BP** and **MTV-MOF/SWCNT-BP** were measured with a Sauter TVO-S tensile tester equipped with a Sauter FH-1k digital dynamometer and AFH FAST software (Sauter GmbH, Balingen, Germany). Rectangular strips (width 10 mm and length 34 mm) were tested at a strain rate of 0.1 mm min^{-1}. The test allowed the determination of the mechanical properties such as the tensile strength as the maximum stress and the Young's modulus (Figure S11).

Pore distribution measurements:

Pore size distribution was evaluated by a capillary flow porometer (CFP-1500 AEXL, PMI Porous Materials Inc., Ithaca, NY, USA). Membranes were fully wetted by keeping them in Porewick® (Sigma-Aldrich, Milan, Italy) for 24 h. Then, nitrogen was gradually allowed to flow into the membrane by increasing its pressure and the registration of gas pressure and permeation flow rate allowed the calculation of the pore size distribution (Figure S8).

X-ray crystallographic data collection and structure refinement: Crystals of **1** and **[Pb(NO$_3$)$_2$]@1** with *ca.* 0.16 x 0.12 x 0.10 and 0.14 x 0.08 x 0.10, mm as dimensions were selected and mounted on a MITIGEN holder in Paratone oil. Measurement on single crystal of **1** was performed at room temperature, whereas single crystal of **[Pb(NO$_3$)$_2$]@1** was very quickly placed on a nitrogen stream cooled at 100 K to avoid the possible degradation upon dehydration. Diffraction data were collected on a Bruker-Nonius X8APEXII CCD area detector diffractometer using graphite-monochromated Mo-K$_\alpha$ radiation (λ = 0.71073 Å) for. The data were processed through SAINT[7] reduction and SADABS[8] multi-scan absorption software. The structure was solved with the SHELXS structure solution program, using the Patterson method. The model was refined with version 2018/3 of SHELXL against F^2 on all data by full-matrix least squares.[9-11]

Crystals of **[Pb(NO$_3$)$_2$]@1**, suitable for X-ray diffraction, were obtained by soaking crystals of **1** (5.0 mg) in a saturated aqueous solution of Pb(NO$_3$)$_2$ for 6 hours, after a crystal-to-crystal transformation. For these reasons, it is reasonable to observe a diffraction pattern sometimes affected by expected internal imperfections of the crystals.

All non-hydrogen were refined anisotropically except some carbon and sulfur atoms belonging to the highly thermal disordered methionine and methylcysteine chains pointing within huge pores and disordered Pb atoms in **[Pb(NO$_3$)$_2$]@1.** In the latter, the occupancy factors, of Pb^{2+} metal ions have been defined in agreement with SEM results. Pb^{2+} metal ions exhibit statistic and sever thermal disorder, as normally expected in porous crystals. Indeed, the heavy metal ion is detected residing either far (Pb1A with assigned occupancy factor of 0.08333) or close to the methionine moieties (Pb1 and Pb1B with occupancy factors of 0.16777 and 0.08333, respectively), quite suggesting a pre- and post-recognition from the aminoacidic derivative, with Pb1B representing the first step of migration of Pb1A (the most far from the walls of the MTV-MOF) towards the final site of Pb1, where the Pb^{2+} metal ions interacts both with sulfur atom belonging from methionine derivative and oxygen atoms from the oxamate ligand (Figure S2).

In both crystal structure refinements, the use of some C-C and C-S together with Pb-S bond, in **[Pb(NO$_3$)$_2$]@1**, lengths restrains during the refinements of some highly disordered atoms, has been reasonable imposed and related to ethyl- and methyl-thiomethyl chains of the methox and mecysmox ligands, respectively that are dynamic components of the frameworks. As a consequence Alerts A and B in the checkcifs, also related to short intra H···H, are detected.

The solvent molecules in both models, together with NO_3^- anions expected in **[Pb(NO$_3$)$_2$]@1**, were disordered and no reasonable models have been found to define them. For that reason, the contribution to the diffraction pattern from the disordered water molecules located in the voids was subtracted from the observed data through the SQUEEZE method, implemented in PLATON.[12] The hydrogen atoms of the ligand were set in calculated positions and refined as riding atoms whereas for water molecules were neither found nor calculated.

A summary of the crystallographic data and structure refinement for **1** and **[Pb(NO$_3$)$_2$]@1** is given in Table S1. The comments for the alerts A and B are reported in the CIFs using the validation response form (vrf). The somewhat high R values (levels Alert A and B in checkcifs) is, most likely, affected by the contribution of the highly disordered solvent and anions to the intensities of the low angle reflections. CCDC (Cambridge Crystallographic Data Centre) reference numbers are 2128259-2128260 for **1** and **[Pb(NO$_3$)$_2$]@1**, respectively.

The final geometrical calculations on free voids and the graphical manipulations were carried out with PLATON[12] implemented in WinGX,[13] and CRYSTAL MAKER[14] programs, respectively.

Table S1. Summary of Crystallographic Data for **1** and **[Pb(NO$_3$)$_2$]@1.**

Compound	**1**	**[Pb(NO$_3$)$_2$]@1**
Formula	$C_{33}H_{122}CaCu_6N_6O_{59}S_6$	C_{33} H_{60} Ca Cu_6 N_8 O_{34} Pb S_6
M (g mol^{-1})	2161.04	1933.76
λ (Å)	0.71073	0.71073
Crystal system	hexagonal	hexagonal
Space group	$P6_3$	$P6_3$
a (Å)	17.8511(7)	17.866(2)
c (Å)	12.8014(8)	12.6684(18)
V (Å^3)	3532.8(4)	3502.0(10)
Z	2	2
ρ_{calc} (g cm^{-3})	2.032	1.834
μ (mm^{-1})	2.154	4.520
T (K)	296	100
θ range for data collection (°)	1.317- 24.620	2.28- 26.45
Completeness to θ = 25.0	94%	99.5%
Measured reflections	37242	59514
Unique reflections (Rint)	3565(0.0469)	4784(0.1047)
Observed reflections [$I > 2\sigma(I)$]	2653	3398
Goof	1.199	1.163
R^a [$I > 2\sigma(I)$] (all data)	0.0615 (0.0811)	0.0773 (0.0998)
wR^b [$I > 2\sigma(I)$] (all data)	0.1762 (0.1840)	0.2198 (0.2352)
CCDC	2128259	2128260

^{a} R = $\sum(|F_o| - |F_c|)/\sum|F_o|$. ^{b} wR = $[\sum w(|F_o| - |F_c|)^2/\sum w|F_o|^2]^{1/2}$.

Table S2. Selected data[a] from the ICP-MS analyses[b] for the aqueous mother solution during the Pb^{2+} adsorption process by 50 mg of a polycrystalline sample of MOF **1**.

Time (min.)	$[Pb^{2+}]$
0	997
1	798
5	734
10	724
15	634
30	259
45	221
60	134
75	79.9
90	46.6
120	23.8
180	22.7
240	17.45
300	7.89
360	6.59
720	5.45
1440	5.12
4320	4.89

[a] Results are given as µg/L. [b] LOD: 0,012 µg/L

Table S3. Residual Pb^{2+} concentration and recovery percentage (RE%) in the stock solutions of deionized water at an initial concentration of about 300 ppb analysed with the ICP-MS for a neat **SWCNT-BP** and **MTV-MOF/ SWCNT-BP**.

**Deionized water**	**300 ppb**					
	**SWCNT-BP**			**MTV-MOF/ SWCNT-BP**		
**Time**	**^{208}Pb**	**SD**	**RE%**	**^{208}Pb**	**SD**	**RE%**
**min**	**ppb**	**ppb**		**ppb**	**ppb**	
0	287.87	3.39	0.00	285.51	0.80	0.00
1	283.73	2.16	1.44	278.09	1.71	2.60
3	278.53	1.40	3.24	271.39	1.65	4.95
5	274.72	0.31	4.57	266.66	0.84	6.60
7	268.98	0.83	6.56	266.54	1.64	6.64
10	266.37	1.70	7.47	254.68	1.76	10.80
15	247.31	2.42	14.09	248.15	2.52	13.09
20	237.69	1.61	17.43	224.30	4.54	21.44
30	203.85	3.81	29.19	190.13	0.95	33.41
45	172.14	1.60	40.20	150.60	1.59	47.25
60	144.05	1.03	49.96	125.14	0.85	56.17
120	54.77	1.37	80.97	35.97	0.14	87.40
240	13.68	0.18	95.25	5.51	0.04	98.07
480	10.74	0.10	96.27	4.67	0.07	98.36
1000	10.50	0.03	96.35	0.49	0.00	99.83
1440	2.75	0.01	99.05	0.27	0.28	99.90
4320	0.74	0.02	99.74	0.10	0.11	99.97

LOD: 0.015 ppb

Table S4. Residual Pb^{2+} concentration and recovery percentage (RE%) in the stock solutions of deionized water at an initial concentration of about 1000 ppb analysed with the ICP-MS for a neat **SWCNT-BP** and **MTV-MOF/SWCNT-BP**.

**Deionized water**	**1000 ppb**					
	**SWCNT-BP**			**MTV-MOF/ SWCNT-BP**		
**Time min**	**^{208}Pb ppb**	**SD ppb**	**RE%**	**^{208}Pb ppb**	**SD ppb**	**RE%**
0	987.61	18.57	0.00	993.21	1.73	0.00
1	941.06	11.23	4.71	936.54	12.93	5.71
3	905.79	8.02	8.28	904.91	8.15	8.89
5	896.23	19.20	9.25	882.24	7.62	11.17
7	870.64	9.37	11.84	855.37	4.69	13.88
10	852.37	6.42	13.69	818.43	7.91	17.60
15	805.98	4.41	18.39	795.13	2.08	19.94
20	760.17	6.96	23.03	720.80	10.74	27.43
30	757.98	8.94	23.25	671.65	6.11	32.38
45	684.44	11.85	30.70	554.84	6.09	44.14
60	603.97	9.13	38.85	468.33	3.28	52.85
120	206.04	2.72	79.14	129.15	0.58	87.00
240	48.25	0.73	95.11	17.41	0.23	98.25
480	39.35	0.43	96.02	14.07	0.05	98.58
1000	6.65	0.06	99.33	0.73	0.02	99.93
1440	5.71	0.07	99.42	0.44	0.01	99.96
4320	4.58	0.11	99.54	0.39	0.12	99.96

LOD: 0.015 ppb

Table S5. Residual Pb^{2+} concentration and recovery percentage (RE%) in the stock solutions of mineral water at an initial concentration of about 300 ppb analysed with the ICP-MS for a neat **SWCNT-BP**. The capture performance was tested also for Na^{+}, Mg^{2+}, K^{+}, and Ca^{2+} ions present in mineral water.

Mineral water 300 ppb – SWCNT-BP											
**Time**	**^{23}Na**		**^{24}Mg**		**^{39}K**		**^{44}Ca**		**^{208}Pb**		
**min**	**ppb**	**SD**	**ppb**	**SD**	**ppb**	**SD**	**ppb**	**SD**	**ppb**	**SD**	**RE%**
0	6871.91	74.56	4920.54	62.81	1312.80	11.39	16637.63	153.02	293.73	3.37	0.00
1	6686.03	123.60	4817.24	140.31	1258.96	20.78	16276.08	111.73	283.42	5.16	3.51
3	6696.62	35.51	4845.49	40.44	1172.87	23.91	16276.09	207.81	280.18	3.47	4.61
5	6529.66	84.87	4696.28	41.69	1141.37	17.84	16104.03	95.72	270.61	4.49	7.87
7	6825.34	46.86	4704.86	32.86	1168.61	15.70	16171.00	83.41	266.30	3.24	9.34
10	6849.81	38.35	4474.49	58.41	1142.06	18.72	15585.43	45.48	257.54	5.18	12.32
15	6690.83	86.43	4516.36	66.16	1184.37	13.33	15542.47	60.13	241.88	3.50	17.65
20	6856.03	182.37	4462.22	84.42	1184.80	22.66	15278.75	294.63	225.28	2.87	23.30
30	6820.05	128.55	4475.45	11.15	1166.94	8.24	14995.49	78.03	195.88	2.09	33.31
45	6775.29	147.37	4535.19	10.59	1179.27	10.68	14830.13	104.73	158.33	1.86	46.10
60	6781.49	150.06	4502.74	56.16	1183.65	21.86	14704.52	136.84	133.19	1.03	54.66
120	6320.56	90.84	4433.10	56.74	1175.66	20.20	14545.28	104.93	62.95	0.64	78.57
240	6181.54	135.54	4589.12	14.02	1231.38	10.33	13749.16	209.70	22.94	0.15	92.19
480	6378.30	60.29	4327.91	112.74	1174.71	15.26	13550.60	73.28	20.60	0.13	92.99
1000	6569.74	73.00	4615.92	69.67	1246.08	21.73	13514.85	80.31	2.86	0.03	99.03
1440	6394.11	56.54	4573.10	66.84	1219.59	5.83	13362.88	194.31	2.73	0.03	99.07
4320	6396.42	144.35	4628.81	45.84	1251.99	15.35	13569.02	196.08	0.78	0.01	99.74

LOD: 0.015 ppb

Table S6. Residual Pb^{2+} concentration and recovery percentage (RE%) in the stock solutions of mineral water at an initial concentration of about 300 ppb analysed with the ICP-MS for **MTV-MOF/SWCNT-BP**. The capture performance was tested also for Na^{+}, Mg^{2+}, K^{+}, and Ca^{2+} ions present in mineral water.

**Mineral water 300 ppb – MTV-MOF/SWCNT-BP**											
**Time**	**^{23}Na**		**^{24}Mg**		**^{39}K**		**^{44}Ca**		**^{208}Pb**		
**min**	**ppb**	**SD**	**ppb**	**SD**	**ppb**	**SD**	**ppb**	**SD**	**ppb**	**SD**	**RE%**
0	6712.21	97.47	5099.90	152.52	1668.04	47.45	16921.10	398.73	296.37	4.90	0.00
1	6645.32	160.28	4910.93	155.57	1632.23	50.82	16757.20	220.81	285.51	6.34	3.66
3	6556.25	179.76	4963.62	104.98	1645.33	27.58	16711.04	142.73	281.34	1.24	5.07
5	6567.21	199.20	4930.48	18.26	1684.23	51.67	17051.17	369.40	267.25	3.75	9.82
7	6544.19	134.17	4953.54	106.24	1558.51	11.05	16552.03	318.01	263.57	2.39	11.07
10	6564.92	155.52	4967.77	152.24	1550.63	41.09	16855.55	559.17	252.90	2.89	14.67
15	6571.14	91.39	4958.22	131.60	1498.53	20.04	16433.20	592.65	243.98	1.79	17.68
20	6438.77	71.66	4883.28	63.51	1487.36	22.81	16494.30	366.46	219.48	2.95	25.95
30	6370.28	79.03	4859.73	32.38	1469.68	18.52	16089.10	632.35	189.10	2.37	36.19
45	6313.65	174.78	4737.96	85.99	1365.41	15.99	15476.38	519.60	128.78	0.70	56.55
60	6225.80	72.15	4660.93	78.76	1321.21	14.75	15818.71	411.04	90.98	1.23	69.30
120	6578.66	99.14	4429.78	50.78	1388.73	18.91	15561.95	653.22	54.42	0.38	81.64
240	6452.84	118.17	4337.49	21.50	1362.32	13.57	15052.70	572.06	16.89	0.20	94.30
480	6539.24	382.08	4323.51	80.39	1347.35	5.02	13224.44	445.71	15.14	0.23	94.89
1000	6548.91	119.05	4506.08	26.87	1384.41	27.31	15109.03	270.32	2.24	0.03	99.24
1440	6458.55	118.87	4476.48	67.19	1372.32	11.36	14859.53	445.20	1.88	0.04	99.37
4320	6248.34	150.92	4507.52	101.03	1332.31	4.86	13416.08	580.81	0.01	0.01	100.00

LOD: 0.015 ppb

Table S7. Residual Pb^{2+} concentration and recovery percentage (RE%) in the stock solutions of mineral water at an initial concentration of about 1000 ppb analysed with the ICP-MS for a neat **SWCNT-BP**. The capture performance was tested also for Na^{+}, Mg^{2+}, K^{+}, and Ca^{2+} ions present in mineral water.

**Mineral water 1000 ppb – SWCNT-BP**												
**Time**	**^{23}Na**		**^{24}Mg**		**^{39}K**		**^{44}Ca**		**^{208}Pb**			
**min**	**ppb**	**SD**	**ppb**	**SD**	**ppb**	**SD**	**ppb**	**SD**	**ppb**	**SD**	**RE%**
0	6979.91	55.84	4945.72	81.69	1292.22	25.55	16798.67	93.08	997.60	13.97	0.00
1	6789.47	100.03	4872.17	100.06	1218.08	17.52	16354.61	170.79	963.57	16.42	3.41
3	6883.22	50.57	4849.25	75.10	1157.94	15.91	16123.76	198.43	956.32	10.06	4.14
5	6896.39	86.91	4745.64	53.70	1161.52	23.25	16140.85	256.77	924.19	28.82	7.36
7	6793.70	49.07	4698.50	68.83	1203.30	11.67	15598.11	96.72	898.13	12.75	9.97
10	6605.50	40.76	4597.45	32.84	1214.88	19.59	15601.44	186.47	880.13	9.09	11.78
15	6150.97	74.93	4634.09	98.82	1164.80	10.10	15325.99	213.11	851.95	8.92	14.60
20	6124.14	59.71	4507.51	126.89	1197.72	18.78	15184.39	294.63	729.06	6.24	26.92
30	6115.12	28.80	4531.90	37.07	1179.46	21.45	15018.41	52.02	661.01	12.00	33.74
45	6200.12	77.72	4489.67	63.50	1192.81	17.22	14877.18	219.28	594.69	16.85	40.39
60	6152.46	81.09	4510.62	94.03	1219.19	16.67	14699.75	181.66	506.23	13.70	49.26
120	6295.25	83.82	4387.14	112.71	1182.76	12.29	14206.29	63.35	298.87	7.54	70.04
240	6165.90	90.19	4452.68	73.83	1173.46	8.37	13945.91	79.42	143.53	2.10	85.61
480	6064.32	60.29	4477.50	134.77	1215.68	21.82	13789.65	186.46	84.13	1.02	91.57
1000	6176.15	92.70	4541.63	108.66	1198.57	5.62	13419.96	142.31	20.66	0.57	97.93
1440	6059.76	77.06	4581.69	53.35	1205.95	0.83	13267.45	76.31	13.85	0.04	98.61
4320	6258.11	84.12	4704.93	20.30	1236.08	1.38	13308.08	203.07	7.96	0.19	99.20

LOD: 0.015 ppb

Table S8. Residual Pb^{2+} concentration and recovery percentage (RE%) in the stock solutions of mineral water at an initial concentration of about 1000 ppb analysed with the ICP-MS for **MTV-MOF/SWCNT-BP**. The capture performance was tested also for Na^{+}, Mg^{2+}, K^{+}, and Ca^{2+} ions present in mineral water.

**Mineral water 1000 ppb – MTV-MOF/SWCNT-BP**												
**Time**	**^{23}Na**		**^{24}Mg**		**^{39}K**		**^{44}Ca**		**^{208}Pb**			
**min**	**ppb**	**SD**	**Ppb**	**SD**	**ppb**	**SD**	**ppb**	**SD**	**ppb**	**SD**	**RE%**
0	6759.38	158.40	5023.08	78.88	1498.26	20.88	16995.46	175.71	995.39	4.90	0.00
1	6723.76	90.39	4996.97	96.59	1423.04	7.08	16803.50	134.79	958.89	6.34	3.67
3	6606.57	73.25	4958.48	157.72	1482.65	58.66	16745.37	208.77	941.66	1.24	5.40
5	6598.76	142.24	4947.75	124.24	1403.25	36.38	16702.73	219.48	907.68	3.75	8.81
7	6653.03	107.17	4975.52	68.23	1397.56	47.25	16786.03	154.25	843.22	2.39	15.29
10	6701.31	83.85	4921.93	118.15	1503.62	18.01	17102.86	123.16	812.42	2.89	18.38
15	6698.45	135.39	4903.93	47.72	1516.73	56.07	16817.04	196.11	743.96	1.79	25.26
20	6593.12	128.67	4894.24	91.54	1491.81	39.30	16763.26	201.44	665.21	2.95	33.17
30	6505.85	100.20	4821.09	107.42	1512.69	25.17	16549.09	95.41	593.52	2.37	40.37
45	6723.26	54.72	4786.73	189.75	1438.42	10.55	16296.99	159.62	510.35	0.70	48.73
60	6623.60	164.59	4710.59	63.20	1407.22	44.73	16008.58	103.07	412.76	1.23	58.53
120	6597.81	179.48	4608.19	105.82	1356.89	57.24	15397.34	176.91	204.27	0.38	79.48
240	6687.17	120.19	4593.72	78.23	1349.24	35.71	14996.10	84.88	77.57	0.20	92.21
480	6521.32	85.08	4486.57	45.36	1313.58	5.02	14237.76	142.72	46.04	0.23	95.37
1000	6603.98	52.64	4387.01	142.08	1295.73	31.35	14039.04	119.33	6.85	0.03	99.31
1440	6463.27	175.66	4507.44	175.18	1276.52	23.37	13792.54	245.55	3.88	0.04	99.61
4320	6296.84	94.89	4548.16	97.20	1359.20	29.38	13567.03	156.01	1.76	0.01	99.82

LOD: 0.015 ppb

Table S9. Residual Pb^{2+} concentration and recovery percentage (RE%) in the stock solutions of mineral water at an initial concentration of about 200 ppb analysed with the ICP-MS for a neat **SWCNT-BP**. The capture performance was tested also for Na^{+}, Mg^{2+}, K^{+}, and Ca^{2+} ions present in mineral water.

**Mineral water 200 ppb – SWCNT-BP**												
**Time**	**^{23}Na**		**^{24}Mg**		**^{39}K**		**^{44}Ca**		**^{208}Pb**			
**min**	**ppb**	**SD**	**ppb**	**SD**	**ppb**	**SD**	**ppb**	**SD**	**ppb**	**SD**	RE%
0	6676.80	27.39	5108.88	81.08	1356.60	15.53	16960.68	95.47	183.80	1.17	0.00
1	6592.12	176.15	5022.29	75.30	1281.73	20.53	16788.62	278.11	165.67	1.12	9.86
3	6602.57	92.62	5081.92	76.49	1271.88	9.67	16706.35	169.03	163.59	1.36	11.00
5	6566.34	60.11	4998.03	66.31	1262.21	6.97	15574.14	6.83	158.43	1.25	13.80
7	6729.48	67.63	5067.68	63.21	1285.21	14.21	15670.73	102.46	155.58	1.53	15.35
10	6753.60	76.72	4952.52	64.60	1315.88	5.16	15517.24	84.19	150.14	2.10	18.32
15	6690.83	72.74	4831.15	86.20	1319.48	25.01	15212.41	198.55	142.01	1.83	22.74
20	6759.74	46.64	4867.90	106.42	1323.65	17.55	14993.35	147.56	129.74	0.75	29.41
30	6724.26	49.40	4905.12	102.49	1312.99	10.74	14918.65	80.12	112.20	1.05	38.96
45	6737.23	90.83	4857.80	54.98	1323.79	15.72	14687.97	87.54	91.44	1.26	50.25
60	6686.25	206.08	5044.64	39.06	1348.35	14.97	14896.06	82.07	80.98	0.75	55.94
120	6231.79	97.94	4873.00	86.79	1342.09	5.91	14117.46	106.93	36.11	0.45	80.35
240	6268.36	144.86	4892.03	98.21	1317.63	12.92	14951.69	121.23	13.74	0.13	92.52
480	6288.72	77.53	4976.80	32.02	1316.33	9.55	14676.36	25.34	11.71	0.11	93.63
1000	6551.28	49.99	4995.78	35.33	1331.27	23.00	14276.79	31.83	2.20	0.04	98.80
1440	6304.31	168.83	5062.15	37.08	1367.07	14.88	13584.85	97.98	2.45	0.01	98.67
4320	6396.42	95.25	4980.65	28.27	1238.89	7.21	13231.39	90.40	0.09	0.06	99.95

LOD: 0.015 ppb

Table S10. Residual Pb^{2+} concentration and recovery percentage (RE%) in the stock solutions of mineral water at an initial concentration of about 200 ppb analysed with the ICP-MS for **MTV-MOF/SWCNT-BP**. The capture performances have been tested also for Na^{+}, Mg^{2+}, K^{+}, and Ca^{2+} ions present in mineral water.

**Mineral water 200 ppb – MTV-MOF/SWCNT-BP**												
**Time**	**^{23}Na**		**^{24}Mg**		**^{39}K**		**^{44}Ca**		**^{208}Pb**			
**min**	**ppb**	**SD**	**ppb**	**SD**	**ppb**	**SD**	**ppb**	**SD**	**ppb**	**SD**	**RE%**
0	6923.33	87.34	4918.22	21.94	1287.33	20.17	17139.60	166.35	186.99	1.42	0.00
1	6579.31	96.32	4828.29	26.02	1236.15	26.19	16854.80	73.82	164.35	3.10	12.11
3	6418.45	115.98	4858.95	79.42	1264.22	16.01	16567.79	171.33	162.16	0.71	13.28
5	6376.71	27.72	4717.94	46.62	1227.42	5.67	16682.78	68.22	148.63	1.49	20.51
7	6265.34	114.17	4718.36	87.97	1259.80	27.32	16204.42	224.92	146.20	1.69	21.81
10	6419.24	98.33	4770.07	76.76	1264.72	15.16	16977.31	227.45	139.52	0.99	25.39
15	6332.51	79.84	4694.76	58.54	1262.09	9.42	16767.61	95.20	122.35	1.34	34.57
20	6478.69	190.85	4797.32	31.41	1281.07	5.08	16199.97	137.66	107.08	0.87	42.74
30	6275.34	97.17	4593.38	79.53	1242.04	43.90	16963.21	60.52	79.47	0.69	57.50
45	6323.16	219.89	4627.24	33.93	1259.27	25.99	16257.51	76.89	59.82	0.70	68.01
60	6362.88	63.75	4708.66	91.13	1280.67	2.26	16965.42	464.92	50.32	0.55	73.09
120	6913.36	92.26	4562.43	14.65	1284.77	12.81	16584.59	521.15	24.33	0.23	86.99
240	6988.54	151.57	4571.68	85.95	1276.94	19.78	16519.95	570.09	12.42	0.08	93.36
480	6804.98	101.45	4215.46	52.65	1282.27	7.34	15285.24	446.86	11.26	0.10	93.98
1000	6792.12	83.63	4493.74	37.46	1261.93	4.50	15947.39	706.29	2.49	0.02	98.67
1440	6863.95	155.00	4509.02	15.48	1274.57	5.81	16388.46	396.40	2.15	0.02	98.85
4320	6821.09	242.39	4478.51	67.49	1258.50	40.64	15158.31	847.48	0.00	0.01	100.00

LOD: 0.015 ppb

Table S11. The obtained Lagergren adsorption rate constants, k_1 (min^{-1}), for **SWCNT-BPs** and **MTV-MOF/SWCNT-BPs** at different initial lead concentrations.

ppb	**SWCNT-BPs**	**MTV-MOF/SWCNT-BPs**
300	0.0120 ± 0.0005	0.0138 ± 0.0006
1000	0.0126 ± 0.0008	0.0143 ± 0.0008

Table S12. Adsorption capacities (AC / mg g^{-1}) and recovery percentage (RE%) by neat **SWCNT-BP** and **MTV-MOF/SWCNT-BP** in 10 - 100 ppm Pb^{2+} range.

Pb^{2+}	SWCNT-BP		MTV-MOF/SWCNT-BP	
**ppm**	**AC / mg g^{-1}**	**RE%**	**AC / mg g^{-1}**	**RE%**
10	52.00	89.76	57.00	98.15
20	77.00	85.2	97.00	97.1
30	121.00	79.15	145.00	93.91
60	128.00	42.23	192.00	65.5
100	180.00	37.49	310.00	64.31

LOD: 0.015 ppb

Table S13. Residual Pb^{2+} concentration and recovery percentage (RE%) in the stock solutions of deionized water at an initial concentration of about 10 ppm analysed with the ICP-MS for a neat **SWCNT-BP** and **MTV-MOF/SWCNT-BP**.

**Deionized water**	**10 ppm**					
	**SWCNT-BP**			**MTV-MOF/ SWCNT-BP**		
**Time min**	**^{208}Pb ppb**	**SD ppb**	**RE%**	**^{208}Pb ppb**	**SD ppb**	**RE%**
0	11681.37	66.93	0	11667.17	154.80	0
1	11489.84	296.53	1.64	11126.69	46.51	4.63
5	11377.41	165.23	2.60	10653.61	108.12	8.69
10	10803.50	17.08	7.52	9639.33	38.18	17.38
15	10278.70	30.77	12.01	8886.27	78.57	23.84
30	9296.89	75.52	20.41	8332.94	64.58	28.58
45	8282.59	58.35	29.10	7925.47	109.90	32.07
60	7373.06	83.56	36.88	6798.52	29.61	41.73
120	5059.86	20.45	56.68	4341.44	49.35	62.79
240	2734.64	31.57	76.59	1977.40	16.59	83.05
480	2194.72	45.24	81.21	1288.87	37.13	88.95
1000	1564.35	27.45	86.61	470.99	4.06	95.96
1440	1263.71	9.27	89.18	331.79	2.19	97.16
4320	1195.88	31.67	89.76	215.35	3.40	98.15

LOD: 0.015 ppb

Table S14. Residual Pb^{2+} concentration and recovery percentage (RE%) in the stock solutions of deionized water at an initial concentration of about 20 ppm analysed with the ICP-MS for a neat **SWCNT-BP** and **MTV-MOF/SWCNT-BP**.

Deionized water	20 ppm					
	SWCNT-BP			MTV-MOF/ SWCNT-BP		
Time min	^{208}Pb ppb	SD ppb	RE%	^{208}Pb ppb	SD ppb	RE%
0	19894.31	103.41	0	20075.97	160.99	0
1	19582.97	98.27	1.56	19189.61	71.63	4.42
5	19057.76	99.93	4.20	18607.41	141.64	7.32
10	18170.47	20.87	8.66	16868.83	34.74	15.98
15	17285.18	41.33	13.11	15931.28	68.36	20.65
30	15868.70	79.66	20.23	14554.07	62.64	27.51
45	14414.43	71.03	27.54	13845.39	108.80	31.04
60	12777.13	87.09	35.77	12470.18	33.16	37.89
120	9158.35	23.26	53.96	8165.90	49.84	59.33
240	6321.42	39.35	68.22	4873.44	18.25	75.73
480	5072.06	56.38	74.50	3072.62	32.30	84.70
1000	3564.07	36.54	82.08	1639.20	13.94	91.84
1440	3261.68	11.55	83.60	1071.05	21.71	94.67
4320	2943.37	32.96	85.20	583.20	13.09	97.10

LOD: 0.015 ppb

Table S15. Residual Pb^{2+} concentration and recovery percentage (RE%) in the stock solutions of deionized water at an initial concentration of about 30 ppm analysed with the ICP-MS for a neat **SWCNT-BP** and **MTV-MOF/SWCNT-BP**.

**Deionized water**	**30 ppm**					
	**SWCNT-BP**			**MTV-MOF/ SWCNT-BP**		
**Time**	**^{208}Pb**	**SD**	**RE%**	**^{208}Pb**	**SD**	**RE%**
**min**	**ppb**	**ppb**		**ppb**	**ppb**	
0	30592.33	170.56	0	30750.21	200.89	0
1	30131.44	149.96	1.51	29467.92	171.87	4.17
5	28849.97	81.74	5.70	28717.62	194.41	6.61
10	27498.93	108.79	10.11	26435.95	91.81	14.03
15	26048.44	32.43	14.85	25018.37	79.85	18.64
30	24411.60	83.95	20.20	22816.65	64.58	25.80
45	22467.36	77.89	26.56	20663.52	99.05	32.80
60	20497.74	96.64	33.00	19836.96	61.81	35.49
120	16121.79	50.44	47.30	13399.70	53.25	56.42
240	12873.25	19.21	57.92	11352.97	39.75	63.08
480	9612.29	25.36	68.58	7235.52	58.29	76.47
1000	8228.39	24.98	73.10	5433.56	16.57	82.33
1440	7779.28	17.39	74.57	3517.82	4.94	88.56
4320	6379.87	40.09	79.15	1872.68	6.79	93.91

LOD: 0.015 ppb

Table S16. Residual Pb^{2+} concentration and recovery percentage (RE%) in the stock solutions of deionized water at an initial concentration of about 60 ppm analysed with the ICP-MS for a neat **SWCNT-BP** and **MTV-MOF/SWCNT-BP**.

**Deionized water**	**60 ppm**					
	**SWCNT-BP**			**MTV-MOF/ SWCNT-BP**		
**Time**	**^{208}Pb**	**SD**	**RE%**	**^{208}Pb**	**SD**	**RE%**
**min**	**ppb**	**ppb**		**ppb**	**ppb**	
0	60613.57	848.74	0	61404.79	335.74	0
1	60084.94	945.96	0.87	59072.51	587.86	3.80
5	59722.59	233.57	1.47	58839.87	700.95	4.18
10	55905.96	548.36	7.77	55240.18	642.84	10.04
15	53100.83	219.80	12.39	52347.70	855.39	14.75
30	48269.77	360.10	20.36	47274.89	395.29	23.01
45	47370.00	316.06	21.85	45705.48	542.69	25.57
60	46804.67	680.68	22.78	44537.52	276.20	27.47
120	45148.24	519.83	25.51	39784.47	119.53	35.21
240	44589.97	296.20	26.44	37576.28	274.82	38.81
480	40889.64	309.04	32.54	36764.41	436.75	40.13
1000	37142.55	480.87	38.72	35121.33	393.62	42.80
1440	36200.65	319.02	40.28	28373.77	163.66	53.79
4320	35019.06	296.90	42.23	23027.28	86.58	65.50

LOD: 0.015 ppb

Table S17. Residual Pb^{2+} concentration and recovery percentage (RE%) in the stock solutions of deionized water at an initial concentration of about 100 ppm analysed with the ICP-MS for a neat **SWCNT-BP** and **MTV-MOF/SWCNT-BP**.

**Deionized water**	**100 ppm**					
	**SWCNT-BP**			**MTV-MOF/ SWCNT-BP**		
**Time**	**^{208}Pb**	**SD**	**RE%**	**^{208}Pb**	**SD**	**RE%**
**min**	**ppb**	**ppb**		**ppb**	**ppb**	
0	95711.39	1188.58	0	96764.29	1282.65	0.00
2880	68080.46	554.53	28.87	42239.43	632.01	56.35
4320	59830.82	523.85	37.49	34535.77	170.12	64.31

LOD: 0.015 ppb

Table S18. Residual concentration and recovery percentage (RE%) in a deionized water solution of Pb^{2+}, Fe^{3+}, and Al^{3+} with an initial concentration of about 1000 ppb for each metal ion, analyzed with the ICP-MS for a neat **SWCNT-BP**.

Deionized water 1000 ppb –SWCNT-BP									
**Time**	**27Al**			**^{57}Fe**			**^{208}Pb**		
**min**	**ppb**	**SD**	**RE%**	**ppb**	**SD**	**RE%**	**ppb**	**SD**	**RE%**
0	1079.88	35.69	0.00	1080.97	69.45	0.00	1003.06	0.84	0.00
1	1030.06	44.28	4.61	1036.74	4.83	4.09	948.20	5.86	5.47
5	967.30	50.18	10.43	1024.97	13.62	5.18	938.28	5.08	6.46
10	943.65	45.58	12.62	1005.34	21.05	7.00	897.30	7.01	10.54
15	929.39	36.07	13.94	987.95	11.49	8.61	819.78	4.13	18.27
30	923.95	53.84	14.44	973.12	9.51	9.98	719.24	3.71	28.30
60	869.36	12.81	19.49	869.21	5.24	19.59	549.81	3.57	45.19
120	605.91	33.41	43.89	862.06	5.80	20.25	213.46	2.64	78.72
240	350.20	23.12	67.57	755.27	7.81	30.13	92.66	0.19	90.76
480	222.99	14.81	79.35	706.14	10.71	34.67	90.03	0.10	91.02
1000	176.15	4.13	83.69	687.24	10.37	36.42	88.92	0.85	91.13
1440	153.99	2.22	85.74	552.13	1.82	48.92	86.68	0.44	91.36
4320	143.08	1.96	86.75	467.85	1.84	56.72	98.11	0.39	90.22

LOD: 0.015 ppb

Table S19. Residual concentration and recovery percentage (RE%) in a deionized water solution of Pb^{2+}, Fe^{3+}, and Al^{3+} with an initial concentration of about 1000 ppb for each metal ion, analyzed with the ICP-MS for a **MOF/SWCNT-BP**.

**Deionized water 1000 ppb – MTV-MOF/SWCNT-BP**									
**Time**	**^{27}Al**			**^{57}Fe**			**^{208}Pb**		
**min**	**ppb**	**SD**	**RE%**	**ppb**	**SD**	**RE%**	**ppb**	**SD**	**RE%**
0	1044.68	91.91	0.00	1003.38	171.95	0.00	1043.58	7.07	0.00
1	997.92	73.21	4.48	985.93	82.07	1.74	961.43	2.07	7.87
5	979.56	54.82	6.23	959.20	74.31	4.40	907.87	1.55	13.00
10	913.31	61.94	12.58	927.83	93.69	7.53	921.52	5.87	11.70
15	898.35	66.86	14.01	904.29	56.13	9.88	876.41	3.65	16.02
30	811.74	71.23	22.30	891.66	78.09	11.13	747.75	2.39	28.35
60	785.52	39.22	24.81	885.09	128.61	11.79	644.26	1.08	38.26
120	782.26	59.82	25.12	877.38	69.38	12.56	436.17	1.87	58.20
240	832.42	18.04	20.32	869.51	29.27	13.34	126.76	1.14	87.85
480	804.39	56.64	23.00	881.60	31.34	12.14	98.72	1.36	90.54
1000	849.08	34.31	18.72	844.93	10.29	15.79	75.84	1.30	92.73
1440	786.61	2.01	24.70	765.54	10.36	23.70	51.96	3.30	95.02
4320	619.76	4.84	40.67	701.51	18.72	30.09	22.91	1.25	97.80

LOD: 0.015 ppb

Table S20: $\log K_D$ values calculated for the lead initial concentrations of 1000 ppb and 10000 ppb in neat and multi-component (Pb, Fe, Al) solutions.

	Neat solution		Multicomponent solution	
**ppb**	**SWCNT-BPs**	**MTV-MOF/SWCNT-BPs**	**SWCNT-BPs**	**MTV-MOF/SWCNT-BPs**
**1000**	5.43	6.50	4.06	4.75
**10000**	4.04	4.82	3.99	4.59

Table S21. Residual concentration and recovery percentage (RE%) in a deionized water solution of Pb^{2+}, Fe^{3+}, and Al^{3+} with an initial concentration of about 10000 ppb for each metal ion, analyzed with the ICP-MS for a neat **SWCNT-BP**.

**Deionized water 10000 ppb – SWCNT-BP**									
**Time**	**^{27}Al**			**^{57}Fe**			**^{208}Pb**		
**min**	**ppb**	**SD**	**RE%**	**ppb**	**SD**	**RE%**	**ppb**	**SD**	**RE%**
0	10894.88	177.03	0.00	10195.31	123.64	0.00	10045.98	189.18	0.00
1	10569.59	167.83	2.99	10023.13	87.05	1.69	9581.26	118.97	4.63
5	10269.54	90.15	5.74	9877.82	142.85	3.11	9215.70	88.51	8.26
10	10109.34	99.40	7.21	9687.12	112.13	4.98	8997.05	107.92	10.44
15	9984.07	81.09	8.36	9436.66	101.42	7.44	8642.69	136.56	13.97
30	9580.62	68.80	12.06	9279.99	79.44	8.98	8349.39	91.71	16.89
60	9003.96	19.13	17.36	9182.30	58.40	9.94	6837.51	87.26	31.94
120	6400.81	36.00	41.25	9101.81	71.23	10.73	4388.23	109.00	56.32
240	3955.54	32.95	63.69	7987.70	56.48	21.65	2351.79	49.87	76.59
480	3785.62	50.79	65.25	7755.67	93.10	23.93	1941.53	51.89	80.67
1000	3660.85	43.60	66.40	7651.04	69.56	24.96	1345.34	89.85	86.61
1440	3226.73	23.41	70.38	5996.67	23.19	41.18	1149.98	46.27	88.55
4320	3511.46	33.65	67.77	5242.40	34.43	48.58	1140.42	41.23	88.65

LOD: 0.015 ppb

Table S22. Residual concentration and recovery percentage (RE%) in a deionized water solution of Pb^{2+}, Fe^{3+}, and Al^{3+} with an initial concentration of about 10000 ppb for each metal ion, analyzed with the ICP-MS for a **MTV-MOF/SWCNT-BP.**

**Deionized water 10000 ppb - MTV-MOF/SWCNT-BP**									
**Time**	**^{27}Al**			**^{57}Fe**			**^{208}Pb**		
**min**	**ppb**	**SD**	**RE%**	**ppb**	**SD**	**RE%**	**ppb**	**SD**	**RE%**
0	9924.42	191.09	0.00	10503.79	119.54	0.00	10435.77	68.69	0.00
1	9694.66	122.08	2.32	10259.29	82.70	2.33	9572.33	65.70	8.27
5	9472.30	58.24	4.56	10012.97	74.09	4.67	9478.74	34.53	9.17
10	9014.75	39.25	9.17	9983.21	61.65	4.96	8932.13	59.18	14.41
15	8444.53	68.60	14.91	9956.92	55.27	5.21	8547.07	66.47	18.10
30	8036.24	102.31	19.03	9616.56	78.95	8.45	7754.54	39.87	25.69
60	7418.42	92.17	25.25	8979.89	126.10	14.51	5688.65	40.79	45.49
120	7806.91	98.24	21.34	8764.83	97.76	16.56	3614.67	28.66	65.36
240	7408.55	80.45	25.35	8495.14	82.70	19.12	1321.64	19.39	87.34
480	7319.93	66.41	26.24	8321.98	53.38	20.77	1180.16	53.60	88.69
1000	7358.10	43.11	25.86	8049.31	22.88	23.37	567.44	20.50	94.56
1440	7158.16	60.09	27.87	7993.41	35.60	23.90	429.62	17.01	95.88
4320	6765.78	48.41	31.83	7702.12	18.25	26.67	327.15	18.48	96.87

LOD: 0.015 ppb

Table S23. Final residual Pb^{2+} concentration and recovery percentage (RE%) in the stock solutions of deionized water at an initial concentration of about 100000 ppb analysed with the ICP-MS for a neat **SWCNT-BP** and **MTV-MOF/SWCNT-BP** after five consecutive use/regeneration cycles. Cycle # 0 represents the initial Pb^{2+} concentration.

**Deionized water**	**100 ppm**					
	**SWCNT-BP**			**MTV-MOF/ SWCNT-BP**		
**Cycle**	**^{208} Pb**	**SD**	**RE%**	**^{208} Pb**	**SD**	**RE%**
**#**	**ppb**	**ppb**		**ppb**	**ppb**	
0	95711.39	1188.58	0	96764.29	1282.65	0
1	59830.82	523.85	37.49	34535.77	1129.08	64.31
2	62224.05	1136.78	34.98	36252.85	1345.10	62.54
3	64530.64	1017.12	32.58	37968.41	1109.76	60.76
4	68802.62	1256.44	28.11	40902.84	1066.28	57.73
5	71756.98	1456.75	25.03	43952.93	1239.76	54.58

LOD: 0.015 ppb

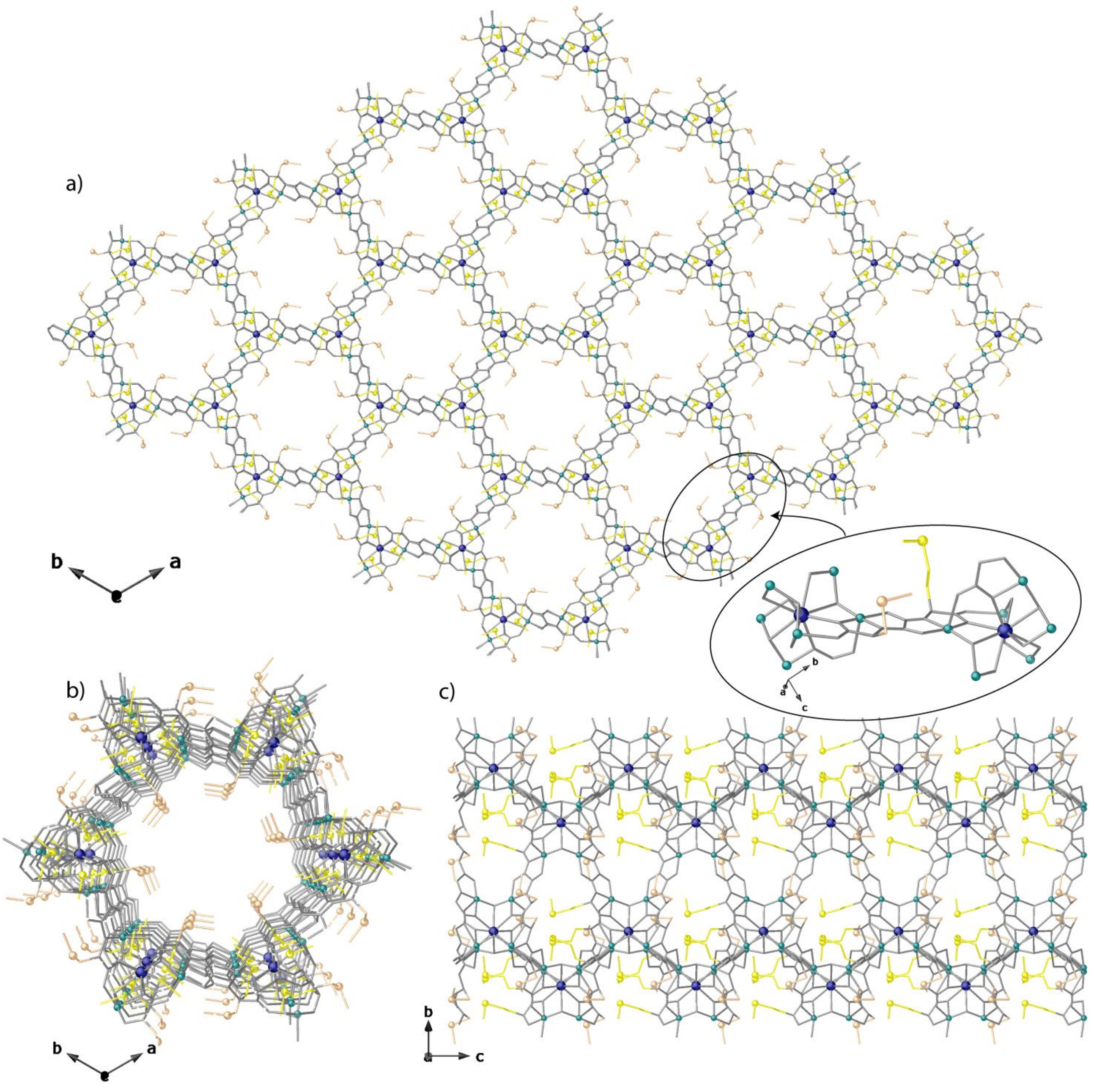

Figure S1. View along *c* crystallographic axis of the porous structure of $\{Ca^{II}Cu^{II}_6[(S,S)$- methox$]_{1.50}[(S,S)$-Mecysmox$]_{1.50}$ $(OH)_2(H_2O)\}$ · $38H_2O$ **1**. (b) Perspective view along *c* crystallographic axis of a single channel of the MTV–MOF **1**. (c) View along [011] direction of a single channel of the crystal structure of MTV–MOF **1**. The crystallization water molecules are omitted for clarity. The inset shows the superimposed snapshot of mixed $\{Cu^{II}_2$[(S,S)-methox/mecysmox]} dimers, on which crystallographic model of **1** is based. Organic ligands are depicted as gray sticks, whereas the amino acid residues are represented with the following color code: $–CH_2SCH_3$ (yellow) and $–CH_2CH_2SCH_3$ (orange). Calcium, copper, and sulfur atoms are shown as blue, cyan, and orange (for methylcysteine fragment)/yellow (for methionine fragment) spheres, respectively.

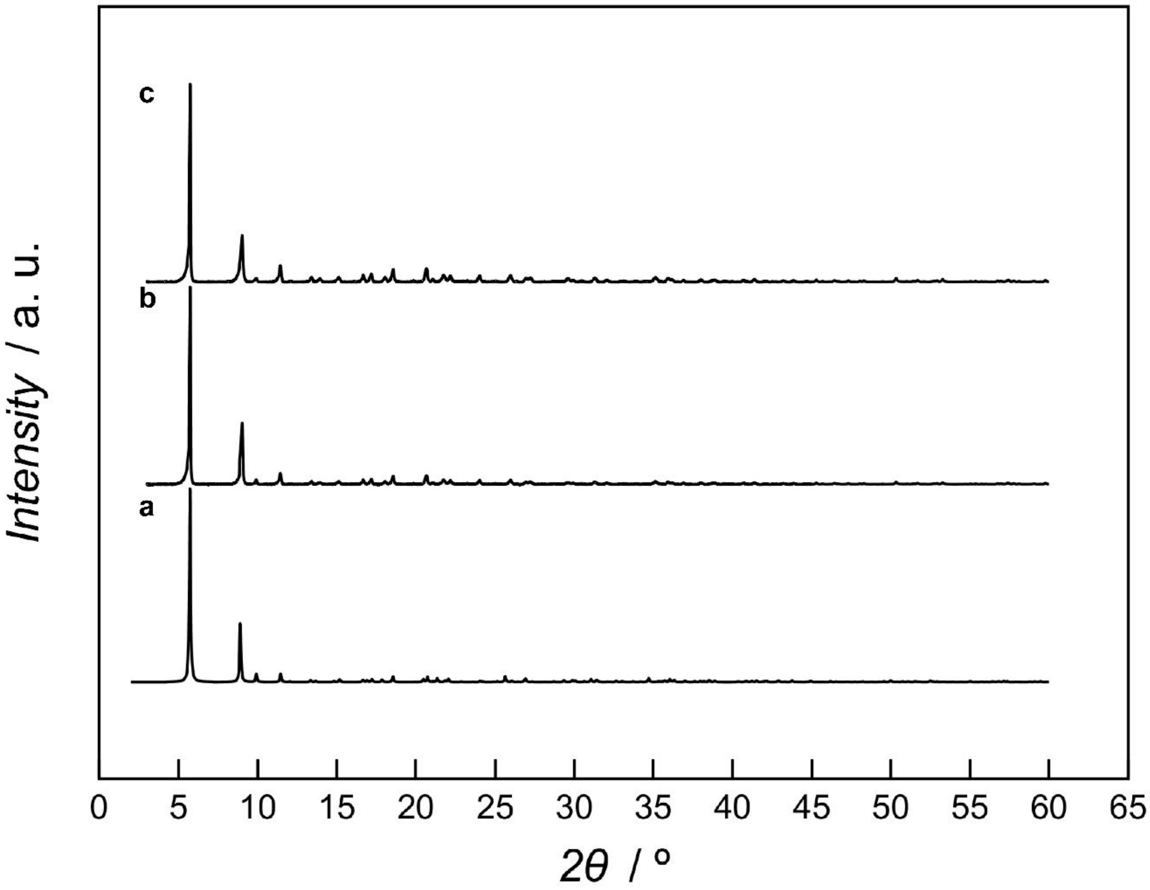

Figure S2. Theoretical (a) and experimental (b) PXRD pattern profiles of **1** in the 2θ range 2–60°. (c) Experimental PXRD pattern profile of **1**, in the 2θ range 2–60°, after the capture experiments with the polycrystalline sample.

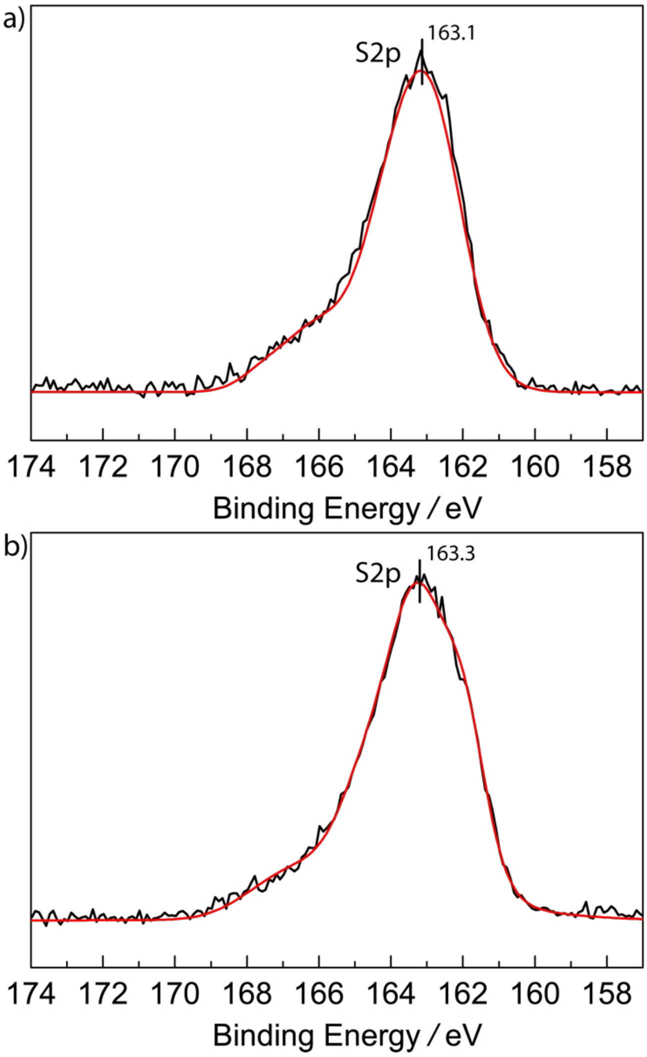

Figure S3. X–ray photoelectron spectroscopy (XPS) of a polycrystalline sample of **1** before (a) and after capture experiments (b).

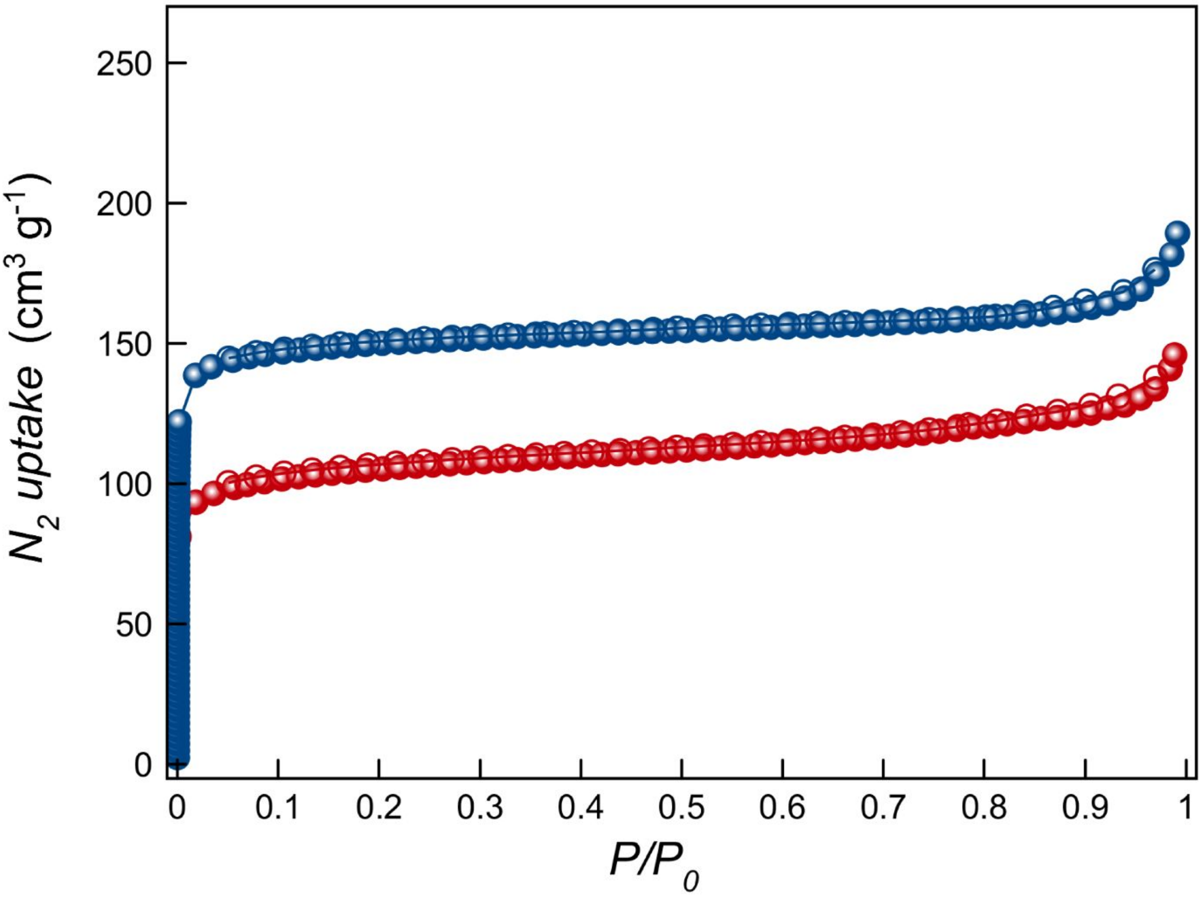

Figure S4. N_2 (77 K) adsorption isotherm for the activated compound **1** before (blue) and after (red) lead capture. Filled and empty symbols indicate the adsorption and desorption isotherms, respectively. The samples were activated at 348 K under reduced pressure for 19 h prior to carry out the sorption measurements.

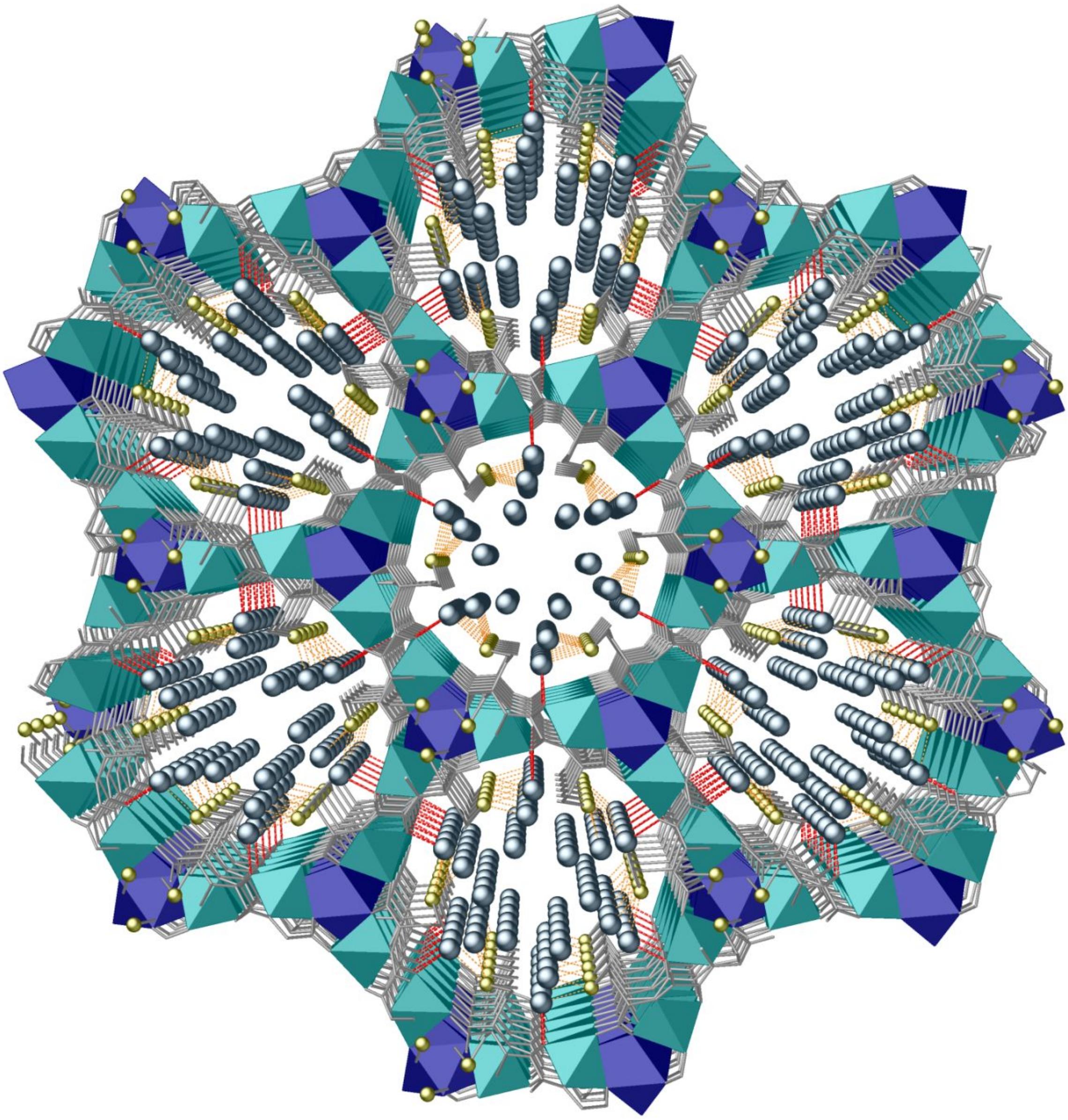

Figure S5. Perspective view along c crystallographic axis of $Pb(NO_3)_2$]@1 crystal structure, showing pores filled by Pb^{2+} metal ions. Color code: copper and calcium atoms from the network are represented by cyan and blue polyhedra, respectively, whereas organic ligands are depicted as grey sticks. Yellow and sky-blue spheres represent S and Pb atoms. S···Pb^{2+} and O···Pb^{2+} interactions are depicted by orange and red dashed lines, respectively.

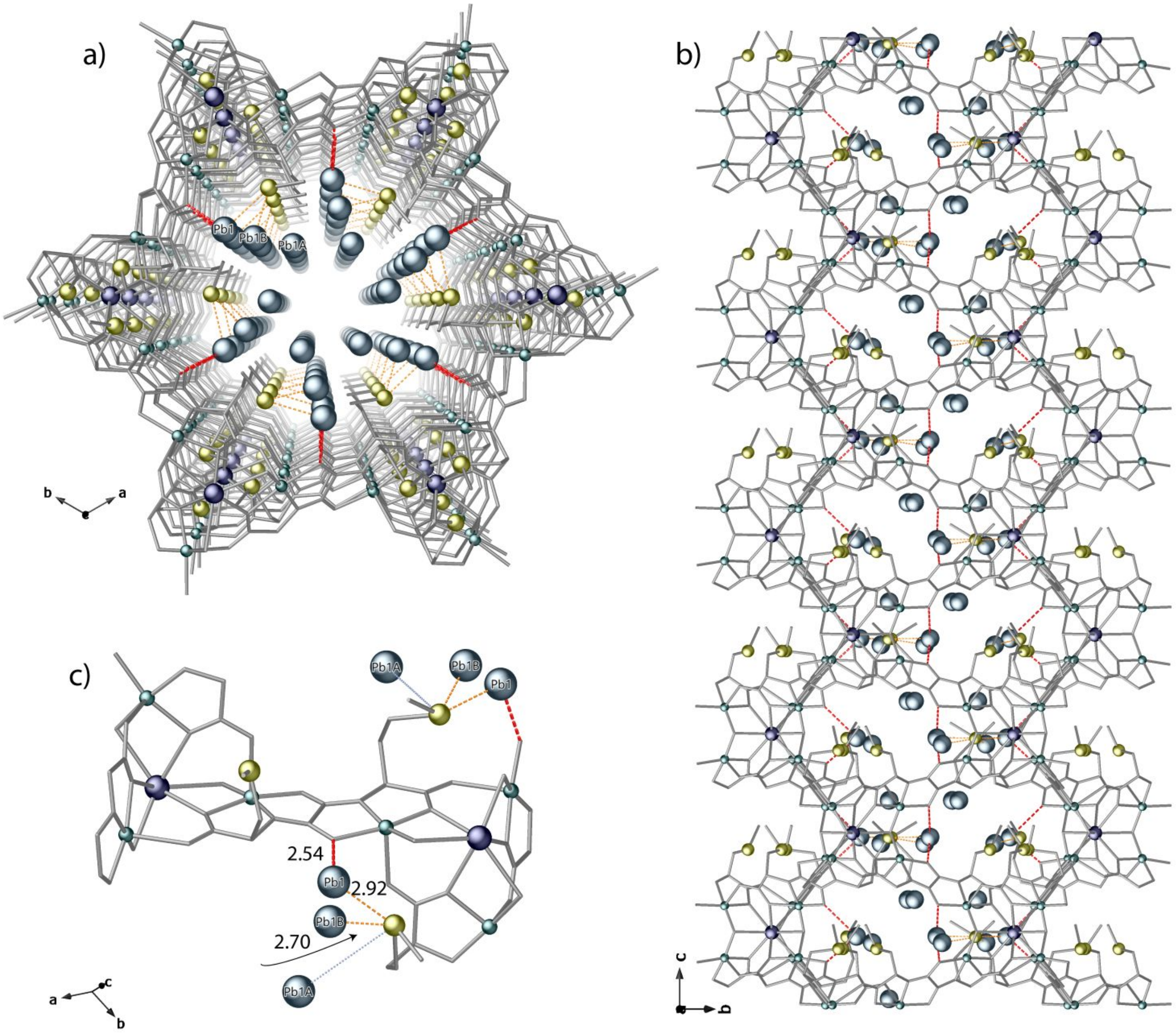

Figure S6. Details of **Pb(NO$_3$)$_2$]@1** crystal structure**:** perspective view along *c* and *a* crystallographic axis of a single pore filled by Pb^{2+} captured metal ions (a) and (b), respectively. (c) Details of Pb^{2+} interactions with sulfur atoms from methionine moieties and oxygen atoms of the oxamate core of the ligand. Color code: copper and calcium atoms from the network are represented by cyan and blue spheres, respectively, whereas organic ligands are depicted as grey sticks. Yellow and sky-blue spheres represent S and Pb atoms. S···Pb^{2+} and O···Pb^{2+} interactions are depicted by blue and red dashed lines, respectively.

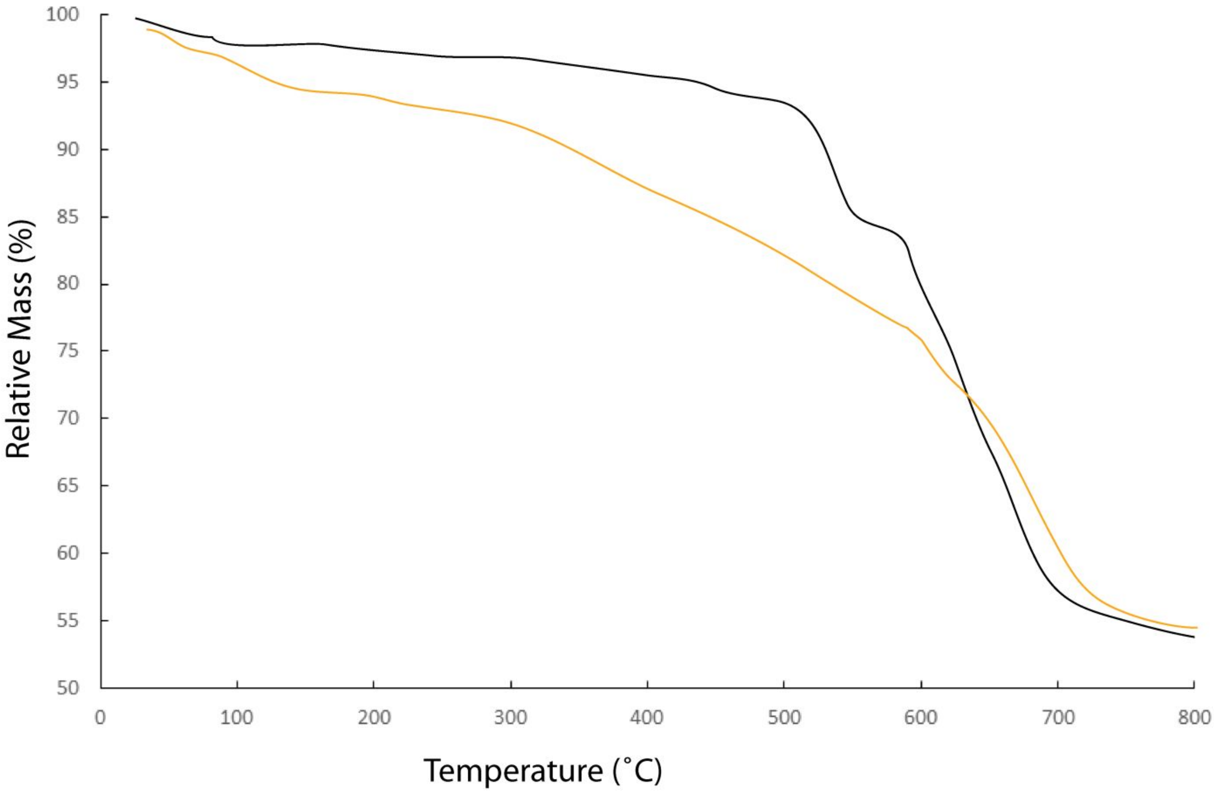

Figure S7. Thermal gravimetrical analyses of a **SWCNT-BP** (black) and a **MTV-MOF/SWCNT-BP** (orange). The initial relative mass change in **MTV-MOF/SWCNT-BP** up to 250 °C is due to solvent loss. The degradation of **SWCNT-BP** and **MTV-MOF/SWCNT-BP** is observed above 450 °C.

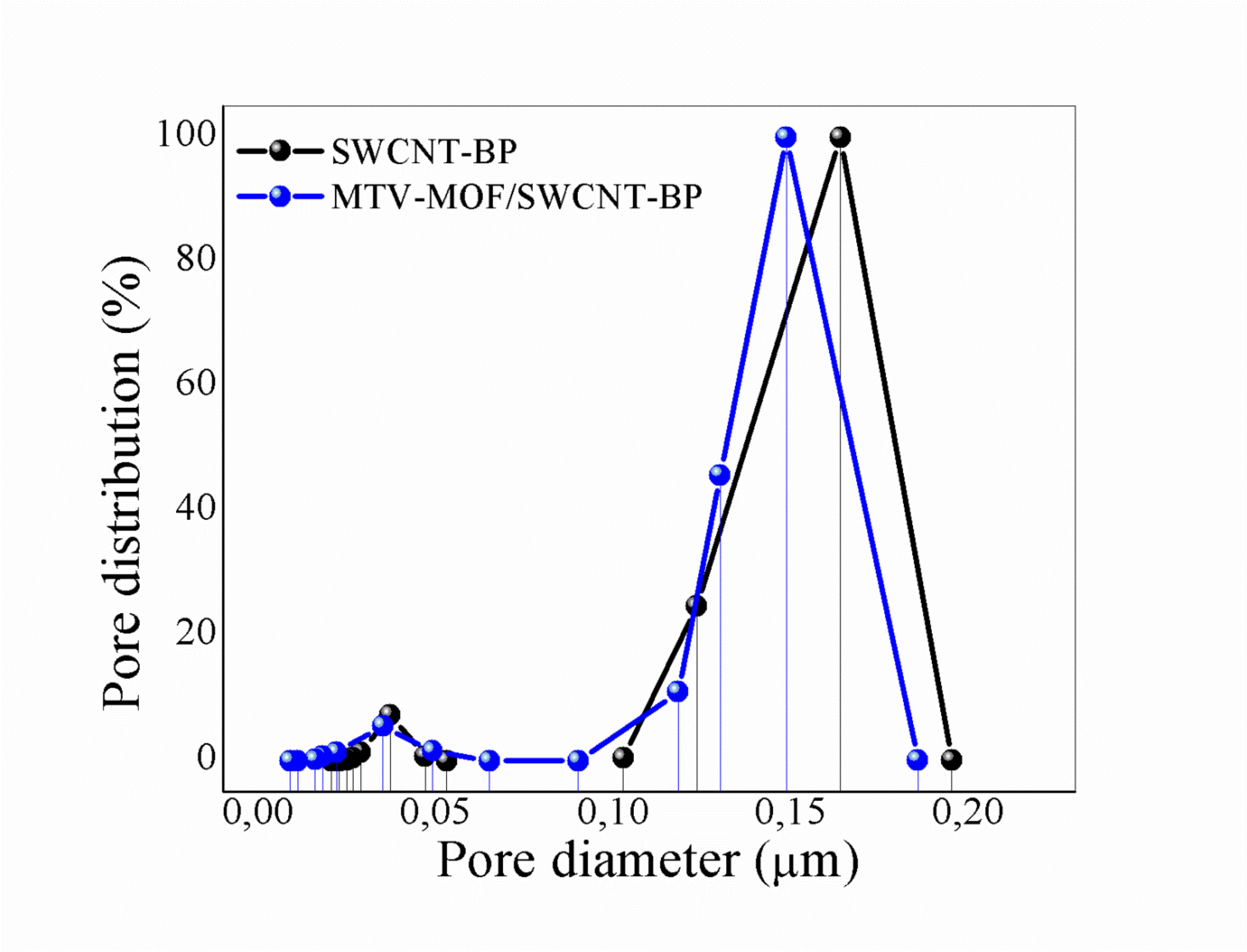

Figure S8. Pores distribution for **SWCNT-BP** and **MTV-MOF/SWCNT** membranes.

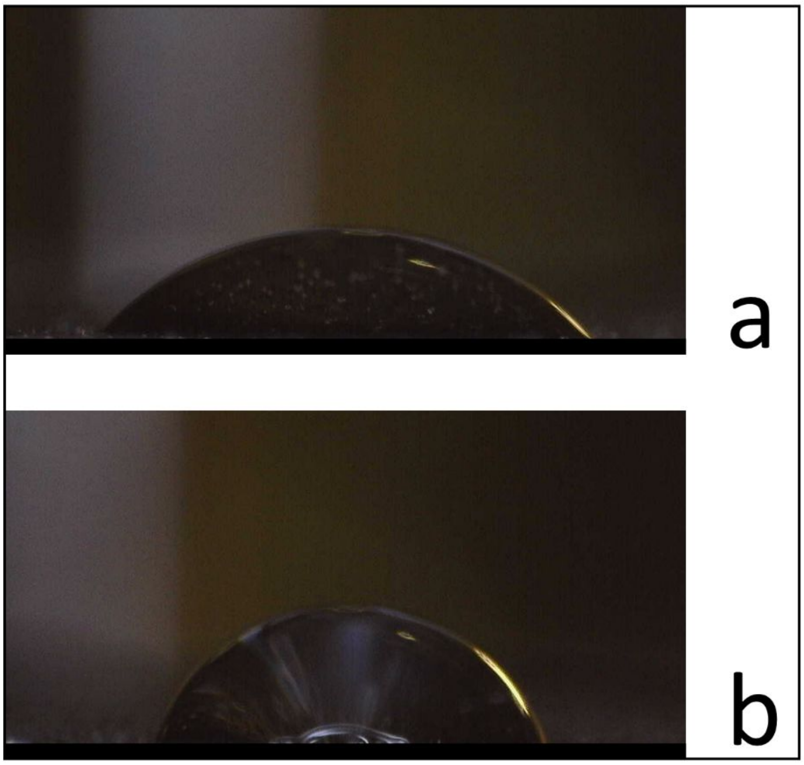

Figure S9. Average contact-angle value of: a) a **SWCNT-BP** (θ = 47.5° ± 0.5°) and b) a **MTV-MOF/SWCNT-BP** (θ = 78.5° ± 0.5°).

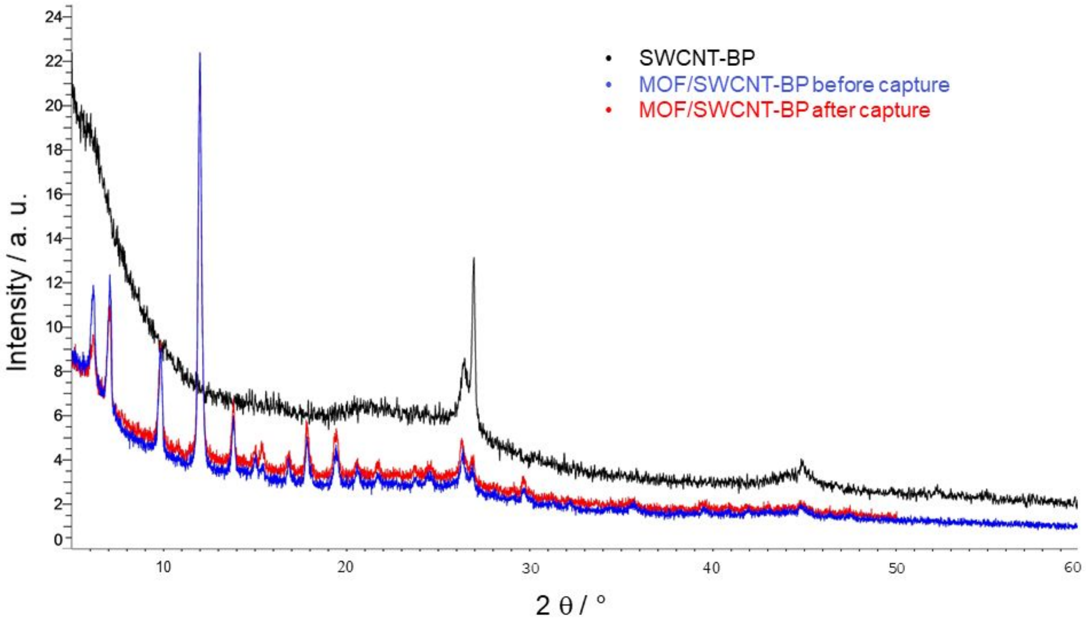

Figure S10. Experimental PXRD pattern profiles of **SWCNT-BP** (black) and **MTV-MOF/SWCNT-BP** membranes before (blue) and after capture and regeneration process (red line) in the 2θ range 2–60°.

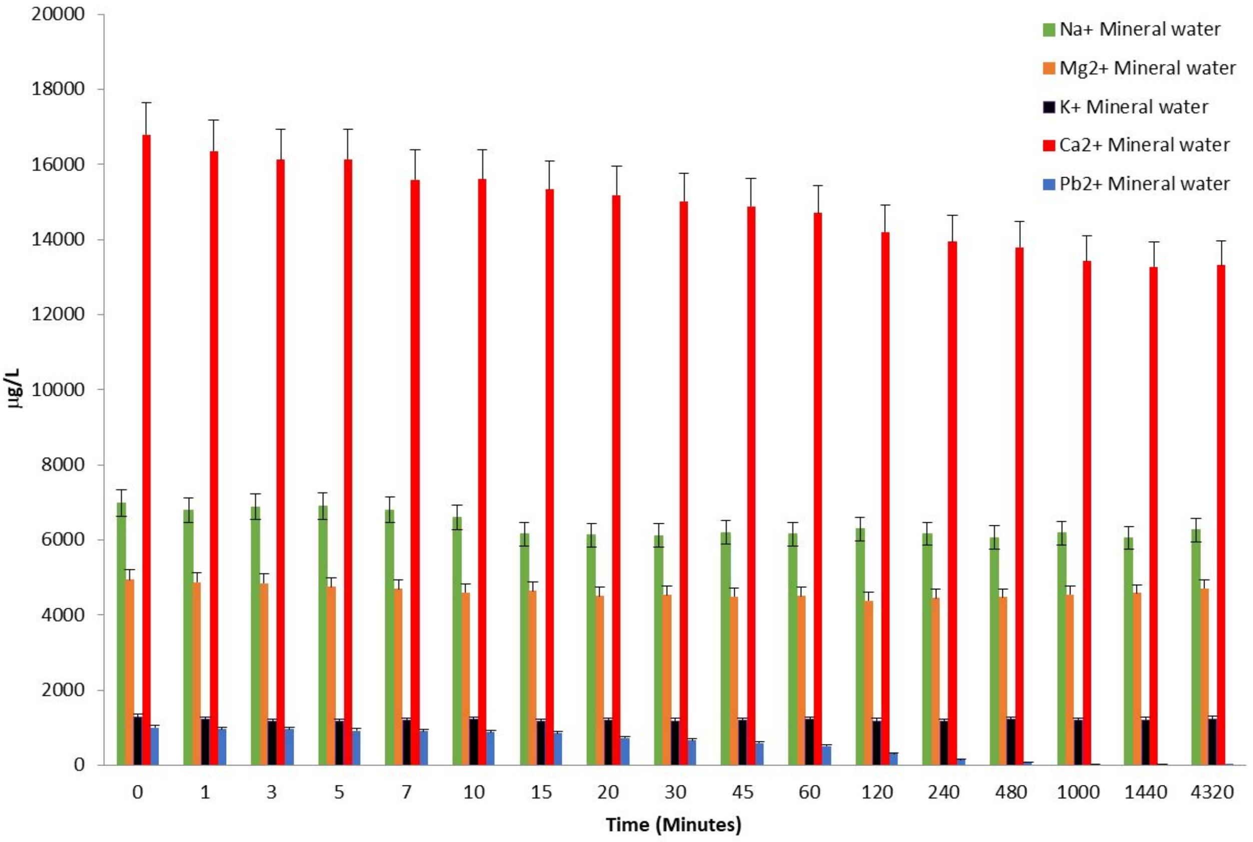

Figure S11. Kinetics and **s**electivity of neat **SWCNT-BP** membrane in mineral water solution with 1000 ppb of [Pb^{2+}]. (Data from Table S7)

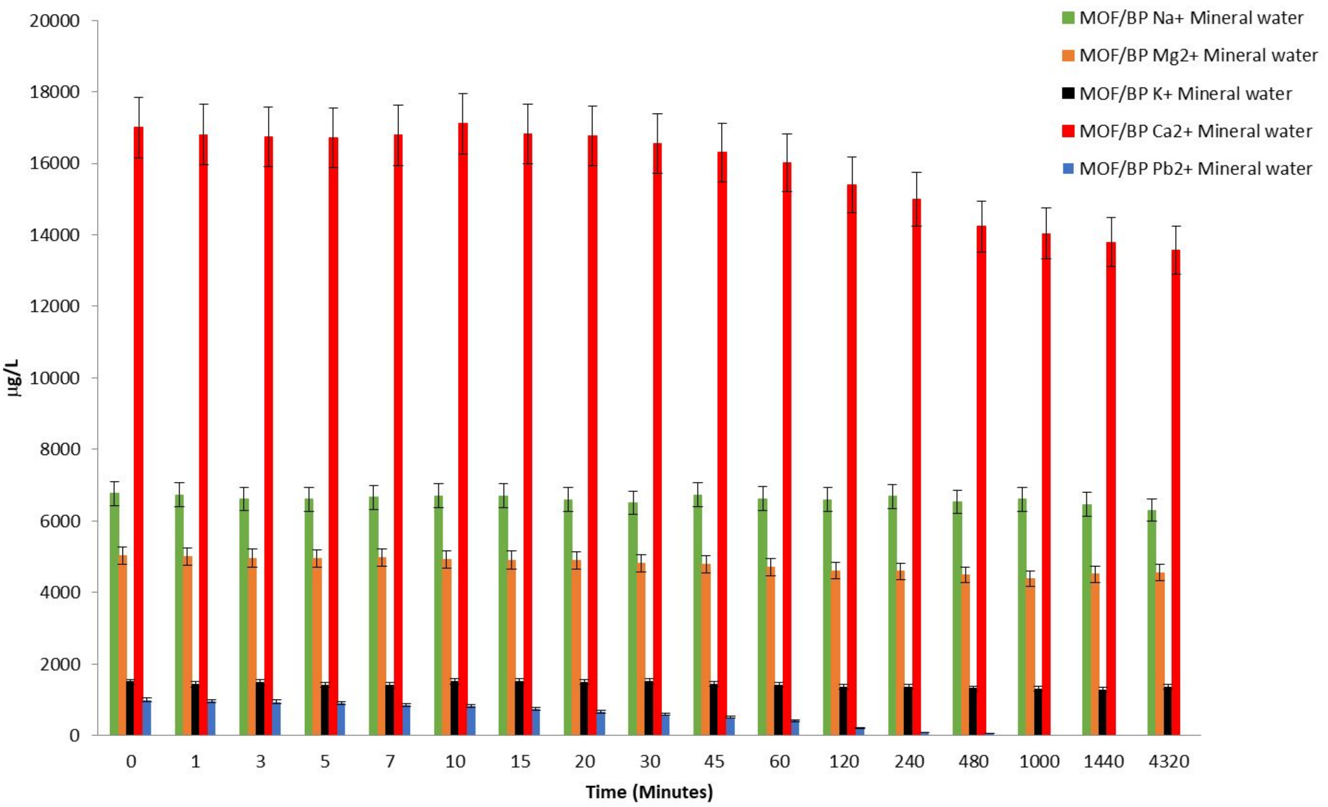

Figure S12. Kinetics and **s**electivity of **MTV-MOF/SWCNT-BP** in mineral water solution with 1000 ppb of [Pb^{2+}]. (Data from Table S8).

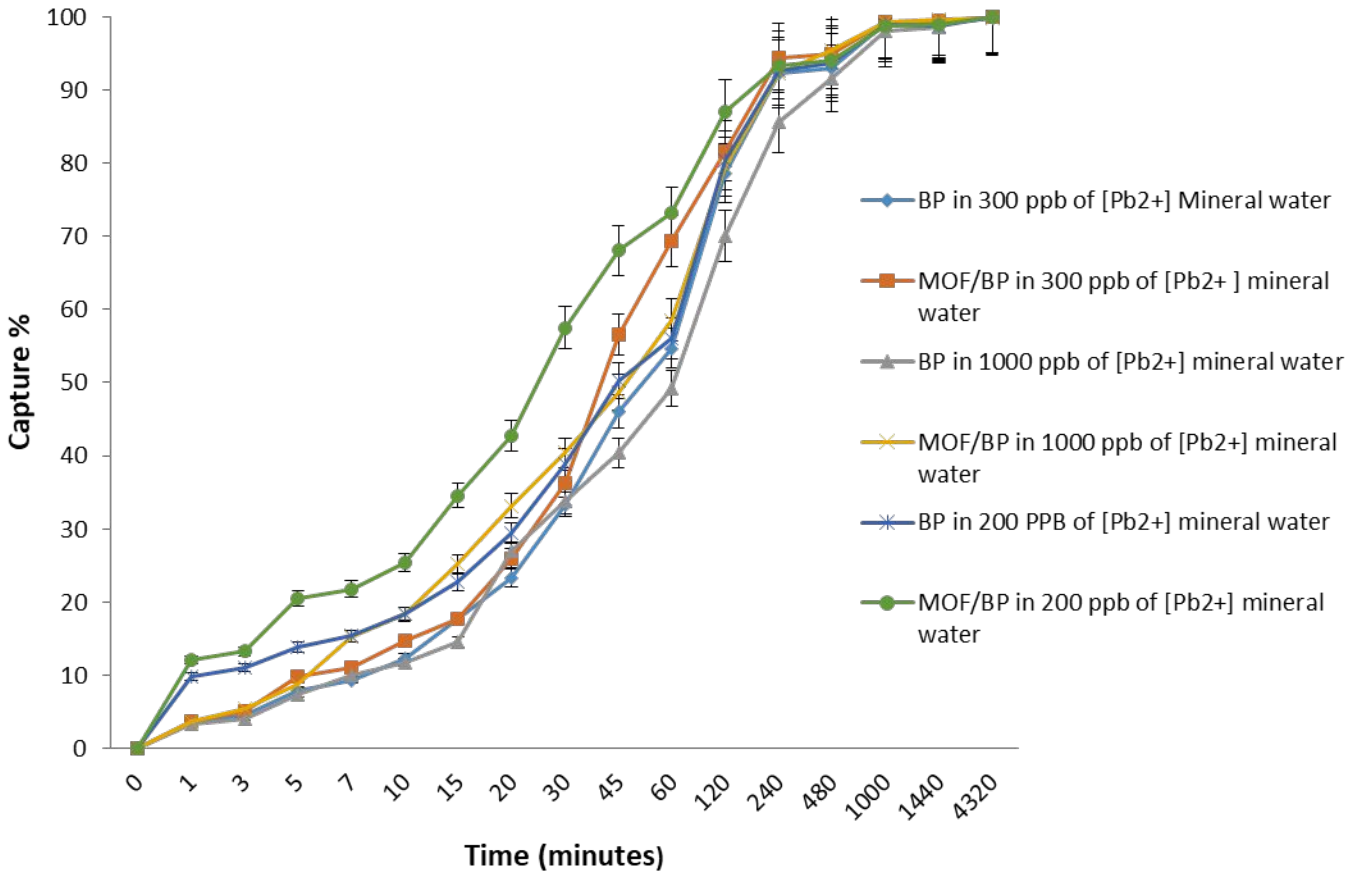

Figure S13. Capture % of Pb^{2+} by **SWCNT-BP** and **MTV-MOF/SWCNT-BP** in mineral water at 200, 300 and 1000 ppb. (Data from Tables S5-S10)

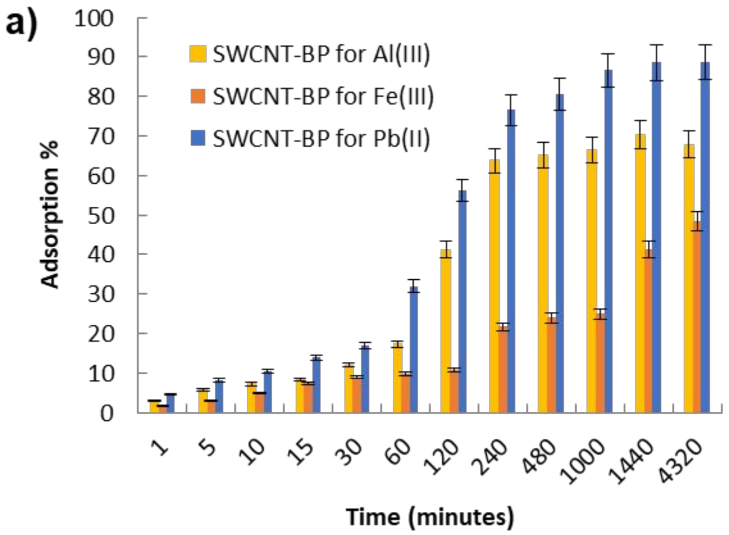

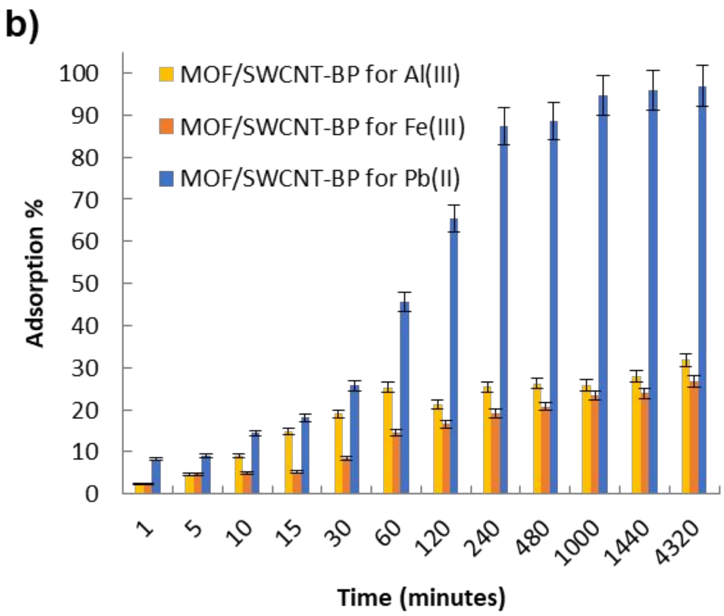

Figure S14. Data for selectivity in 10000 ppb of [Pb^{2+}], [Fe^{3+}], [Al^{3+}] solution for a **SWCNT-BP** (a) and **MTV-MOF/SWCNT-BP** membrane (b) soaked in a volume of 200 mL, in the 0-72 h interval. (Data from Tables S21-S22)

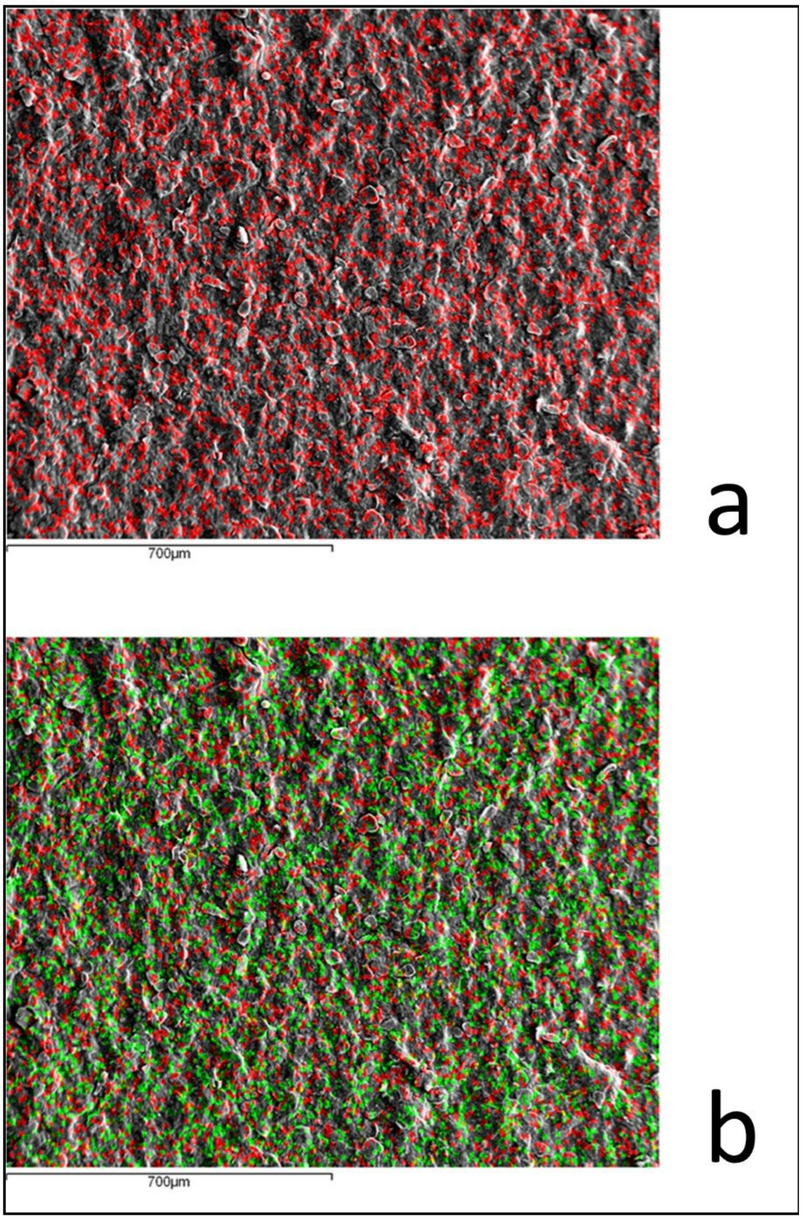

Figure S15. SEM image and the corresponding EDX elemental mapping for Cu and Pb elements from: a) SWCNT-BP and b) a **MTV-MOF/SWCNT-BP** after their use in the capture of Pb(II) (red dots). Green dots in (b) are due to Cu(II) atoms present in **MTV-MOFs**. Images confirm a homogeneous distribution of MTV-MOF in **SWCNT-BP** and a homogeneous adsorption in both membranes of Pb(II).

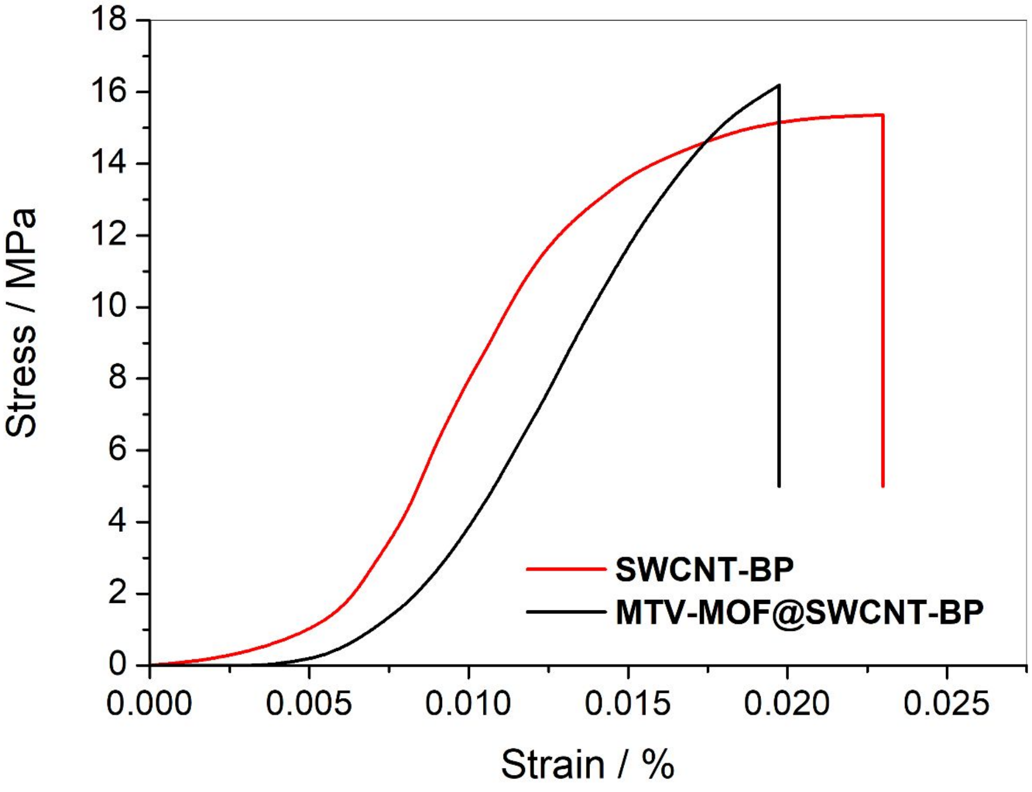

Figure S16. Stress-strain curve for a **SWCNT-BP** and a **MTV-MOF/SWCNT-BP**. The presence of MTV-MOF in SWCNT-BP causes a slight decrease in the Young's modulus from 1.65 ± 0.03 GPa (neat SWCNT-BP) to 1.54 ± 0.02 GPa (**MTV-MOF/SWCNT-BP**). Nevertheless, such magnitudes confirm the high mechanical stability of both membranes.

References:

1. Rahbari, M.; Goharrizi, A. S. Adsorption of Lead(II) from Water by Carbon Nanotubes: Equilibrium, Kinetics, and Thermodynamics. *Water Environment Research* **2009**, *81*, 598-607.

2. Forghani, M.; Azizi, A.; Livani, M.J.; Kafshgari, L. A. Adsorption of Lead(II) and Chromium(VI) from Aqueous Environment onto Metal-Organic Framework MIL-100(Fe): Synthesis, Kinetics, Equilibrium and Thermodynamics. *J. Solid State Chem.* **2020**, *291*, 121636.

3. OriginLab Corporation, Northampton, MA, USA.

4. Belhachemi, M.; Addoun, F. Comparative Adsorption Isotherms and Modeling of Methylene Blue onto Activated Carbons. *Appl. Water Sci.* **2011**, *1*, 111–117.

5. Sedeño-Díaz, J. E.; López-López, E.; Mendoza-Martínez, E.; Rodríguez-Romero, A. J.; Morales-García, S. S. Distribution Coefficient and Metal Pollution Index in Water and Sediments: Proposal of a New Index for Ecological Risk Assessment of Metals. *Water*, **2020**, *12*, 29.

6. US EPA 402-R-99-004A. Understanding Variation in Partition Coefficient, Kd, Values. Volume I - Kd Model, Measurement Methods, and Application of Chemical Reaction Codes. Office of Air and Radiation, Washington DC: USA 1999.

7. SAINT, version 6.45, *Bruker Analytical X-ray Systems, Madison, WI*, 2003.

8. Sheldrick, G. M. SADABS Program for Absorption Correction, version 2.10, *Analytical X-ray Systems, Madison, WI*, 2003

9. Sheldrick, G. M. Crystal structure refinement with SHELXL. *Acta Cryst.* **2015,** *C71,* 3-8.

10. Sheldrick, G. M. A short history of SHELX. *Acta Cryst*. **2008**, *A64*, 112–122.

11. SHELXTL-2013/4, *Bruker Analytical X-ray Instruments, Madison, WI*, 2013.

12. Spek, A. L. Structure validation in chemical crystallography *Acta Cryst*. **2009**, *D65*, 148-155.

13. Farrugia, L. J. WinGX and ORTEP for Windows: An Update. *J. Appl. Crystallogr*. **2012**, *45*, 849–854.

14. Palmer, D. C. Zeitschrift fur Krist. *Cryst. Mater*. **2015**, *230*, 559–572.
